# Reactivity Graph Yields Interpretable IgM Repertoire Signatures as Potential Tumor Biomarkers

**DOI:** 10.3390/ijms24032597

**Published:** 2023-01-30

**Authors:** Dilyan Ferdinandov, Viktor Kostov, Maya Hadzhieva, Velizar Shivarov, Peter Petrov, Assen Bussarsky, Anastas Dimitrov Pashov

**Affiliations:** 1 Clinic of Neurosurgery, St. Ivan Rilski University Hospital, 1431 Sofia, Bulgaria; 2 Stephan Angeloff Institute of Microbiology, Bulgarian Academy of Sciences, 1113 Sofia, Bulgaria; 3 Department of Experimental Research, Medical University—Pleven, 5800 Pleven, Bulgaria; 4 Institute of Mathematics and Informatics, Bulgarian Academy of Sciences, 1113 Sofia, Bulgaria

**Keywords:** IgM repertoire, immunodiagnostics, glioblastoma, CCNB1, HPV, HTLV, IgOme

## Abstract

Combining adaptive and innate immunity induction modes, the repertoire of immunoglobulin M (IgM) can reflect changes in the internal environment including malignancies. Previously, it was shown that a mimotope library reflecting the public IgM repertoire of healthy donors (IgM IgOme) can be mined for efficient probes of tumor biomarker antibody reactivities. To better explore the interpretability of this approach for IgM, solid tumor-related profiles of IgM reactivities to linear epitopes of actual tumor antigens and viral epitopes were studied. The probes were designed as oriented planar microarrays of 4526 peptide sequences (as overlapping 15-mers) derived from 24 tumor-associated antigens and 209 cancer-related B cell epitopes from 30 viral antigens. The IgM reactivity in sera from 21 patients with glioblastoma multiforme, brain metastases of other tumors, and non-tumor-bearing neurosurgery patients was thus probed in a proof-of-principle study. A graph representation of the binding data was developed, which mapped the cross-reactivity of the mixture of IgM (poly)specificities, delineating different antibody footprints in the features of the graph—neighborhoods and cliques. The reactivity graph mapped the major features of the IgM repertoire such as the magnitude of the reactivity (titer) and major cross-reactivities, which correlated with blood group reactivity, non-self recognition, and even idiotypic specificities. A correlation between an aspect of this image of the IgM IgOme, namely, small cliques reflecting rare self-reactivities and the capacity of subsets of the epitopes to separate the diagnostic groups studied was found. In this way, the graph representation helped the feature selection in its filtering step and provided reduced feature sets, which, after recursive feature elimination, produced a classifier containing 51 peptide reactivities separating the three diagnostic groups with an unexpected efficiency. Thus, IgM IgOme approaches to repertoire studies is greatly augmented when self/viral antigens are used and the data are represented as a reactivity graph. This approach is most general, and if it is applicable to tumors in immunologically privileged sites, it can be applied to any solid tumors, for instance, breast or lung cancer.

## 1. Introduction

Any trivial antibody test is used like a keyword search of a log of pathogen encounters. The whole repertoire of antibodies, on the other hand, contains far more information. Attempts to read the repertoire rather as a map of immunological meaning are still rare. The structural studies using antibody repertoire sequencing [1,2,3] have been useful and exciting but still impossible to translate to function at scale. Presently, the workable repertoire-wide functional assays typically use biopanning libraries of peptides on immunoglobulin serum fractions combined with next-generation sequencing (NGS) [4,5,6,7]. Although this new tool provides much greater opportunities, the studies revolve safely in the orbit of the old paradigm, mostly exploring the adaptive immune response to a greater depth. Few groups have tried to extract patterns of reactivities as biomarkers by probing the repertoire with large arrays of peptides or glycans [8,9,10,11], but the deeper questions of the structure of the repertoire is seldom addressed, and the theoretical aspects are still largely unexplored.

The IgM repertoire is a pool of immunochemically largely indistinguishable antibodies that are the product of several B cell compartments [12]. They can be produced for several weeks during the primary T-dependent immune responses (by B2 follicular/extrafollicular cells), in T-independent responses to arrays of, mostly, carbohydrate epitopes (by B2 marginal zone cells), and also constitutively produced with or without the involvement of antigen stimulation (by B1 cells) [13]. They tend (1) to have a low intrinsic affinity, (2) to have a different mutation content with a substantial proportion having few or no somatic mutations, (3) to exhibit higher polyspecificity than IgG, and (4) to often be moderately autoreactive [14]. The cells that produce them are better defined in mice while in humans, these subpopulations have their equivalents of different and sometimes not well-established phenotypes [15,16]. The predominant part (about 80% in mice [17]) of the IgM consists of antibodies with typical properties (produced by B1a not in response to antigens, of few mutations, polyspecific, and moderately autoreactive), and these are called natural autoantibodies (nAAb). On the other hand, the properties of the IgM produced in the primary immune responses and by marginal zone B cells can sometimes overlap functionally with those of nAAb.

The immune system detects tumor-associated antigens (TAA) and appropriately reading the imprint of this information, e.g., in the repertoire changes, could be a source of clinically relevant information [11,18,19]. Several studies have identified both whole proteins and peptides as IgG-targeted antigens that are associated with brain tumors and provide useful clinical information [18,20,21,22]. In all of these studies, the central paradigm is detecting IgG antibody responses to known TAA. Such responses exist in an apparent contradiction to the tenets of immune tolerance to self, as not all antibodies are to mutated (neo)epitopes. On the other hand, there have been arguments in favor of natural/IgM antibodies participating in tumor surveillance [23,24,25], and a hypothesis has been proposed to explain natural antitumor reactivity by quantitative self-tolerance thresholds [24].

With these considerations in view, we previously argued that the repertoire of IgM may be an untapped source of biomarkers [8,26]. It was shown that libraries of mimotopes of public IgM specificities found in the blood of healthy donors may serve as a source of probes to construct diagnostic profiles of IgM reactivities [8,27]. As a proof of principle, such profiles were found to differentiate glioblastoma multiforme (GBM) patients from patients with brain metastases or non-tumor patients. Although peptide mimotopes are structurally interpretable, relating them to actual specificities may be difficult especially in a context of polyspecificity. To gauge the interpretability of IgM reactivity profiles to linear sequences from actual TAA, here we use a similar experimental setting with a commercial planar tumor antigen peptide microarray (PEPperPRINT^®^, Heidelberg, Germany). It contains a sequence set derived from 20 whole-length human proteins known to be TAA as well as 209 viral linear B cell epitopes related to malignancies. Confirming the expected low interpretability of IgM reactivities with self-antigens in the terms of adaptive responses, our results nevertheless identify patterns of IgM reactivity, which generally classify brain tumors. The interpretability of this differential IgM reactivity was found in terms of classes of polyspecificity, mapped using a reactivity graph, and perturbations of the IgM antibody levels.

## 2. Results

### 2.1. The Microarray Binding Data

**Figure 1:**
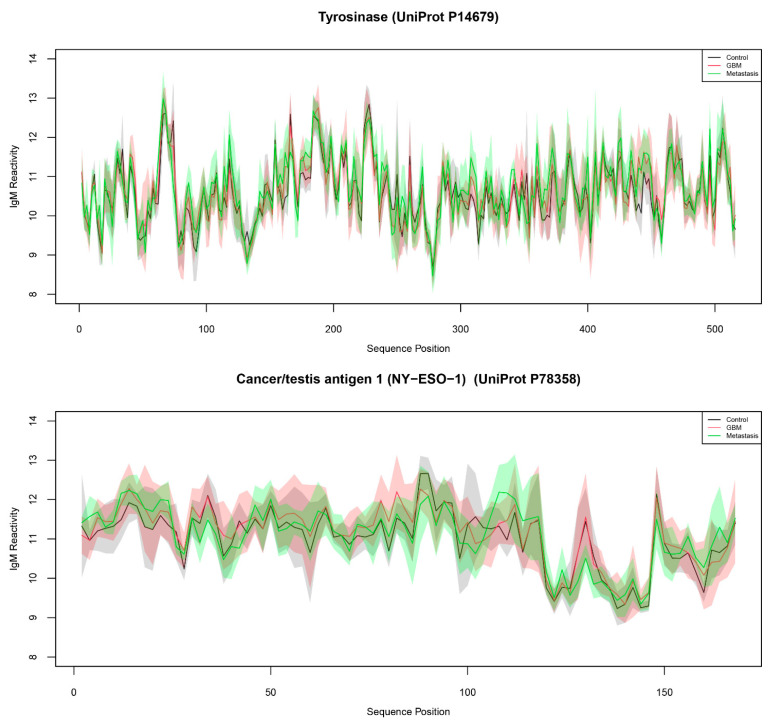
Mean and standard deviation of IgM reactivity from control (black line/grey shading), GBM (red), and metastases (green) patient groups to tyrosinase (**above**) and NY-ESO-1 (**below**). The shaded areas represent the respective standard deviation ranges.

To study the capacity of natural peptides from actual tumor antigens to provide diagnostic IgM reactivity information, we probed a TAA-derived peptide-based microarray with sera from patients with brain tumors and measured the IgM binding. The study included 21 patients divided into three groups according to the diagnosis: histologically proven glioblastoma (GBM, n = 10), cerebral metastases (n = 5, 4 lung cancer, 1 prostate cancer) or control (n = 6). Control subjects were selected randomly among cases with degenerative spine disease. Patients who had undergone previous radio-, chemo- or immunotherapy, who suffered from an autoimmune disease or who had an inflammatory disease in the past three months were excluded. After staining, reading, cleaning, and normalizing for both binding dependence on the amino acid composition (pepStat::normalizeArray function) and for chip effects (limma::normalizeCyclicLoess function), the data represented the IgM reactivities of 21 patients with 4512 peptides. The latter included 24 human tumor antigens scanned with a 15 amino acid frame shifted by two positions and the known linear B cell epitopes from 29 protein antigens of pathogens, mostly viruses (and *H. pylori*) related to cancer (Table 1 and Table 2). The reactivity of IgM with the array showed a considerable correlation between pairs of patients’ sera (r = 0.31–0.84, mean = 0.54) (Supplementary Figure S1). Figure 1 shows typical reactivity with two antigens mapped to the sequence of the proteins. It was very hard to delineate negative reactivity as is typical for IgM, which is highly polyspecific. To assess the interpretability of the IgM profiles, it was necessary to compare the individual peptide reactivities. The traditional statistical approach in microarray data uses the “linear models for microarray data” package limma from Bioconductor. Unfortunately, due to a significant variability in peptide microarray binding, the data seldom yield significant differential expression when based only on the individual reactivities. Indeed, we found only a couple of peptides with significantly different expression between the diagnostic groups. On the other hand, the experimental setting with overlapping peptides is a condition for multiple cross reactivities between the studied peptides. Together with the polyspecificity of the IgM antibodies, these considerations prompt a study of higher-order specificity patterns by correlating the binding to different peptides.

### 2.2. The Reactivity Graph

It has been shown that the complexity of the antibody repertoire is amenable to analysis by graph representations of the relationships between immunoglobulin genes [3] as well as the sequences of the mimotopes in IgOme analysis [27]. Therefore, the cross-reactivities between the set of peptides studied were also represented as a graph.

#### 2.2.1. Construction of the Graph

The mixture of antibodies yielding the binding levels observed in our experiments is unknown. It is conceivable though that the correlation between the patterns of reactivity of two peptides with the sera from a defined set of individuals is a measure of the peptides’ cross-reactivity with antibodies that are related. The presence of a large number of overlapping (11/15 residues) peptide sequences provided a control group of putatively cross-reactive peptide pairs. The Euclidian distance, correlation or cosine distance was considered lossy or inadequate. The ANOVA F statistic proved more informative because it is independent of the global mean level of the compared pair of profiles but is affected by the difference in the mean levels of the pair. Therefore, it was adopted as a similarity measure although it is not a true metric.

**Figure 2:**
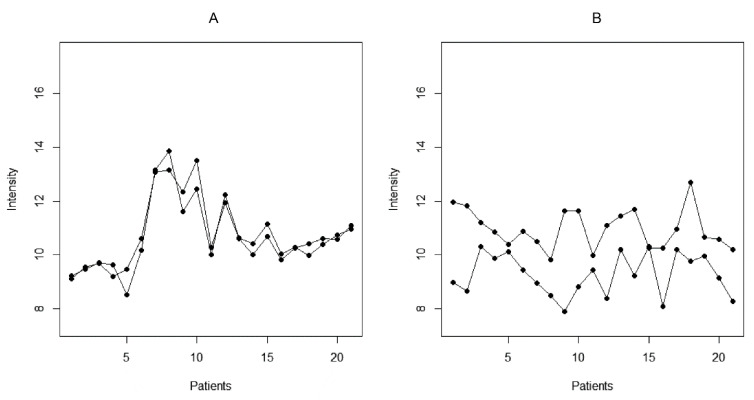
Illustration of cross-reactive (**A**) and dissimilar (**B**) peptide reactivities based on the studied set of 21 patients.

**Figure 3:**
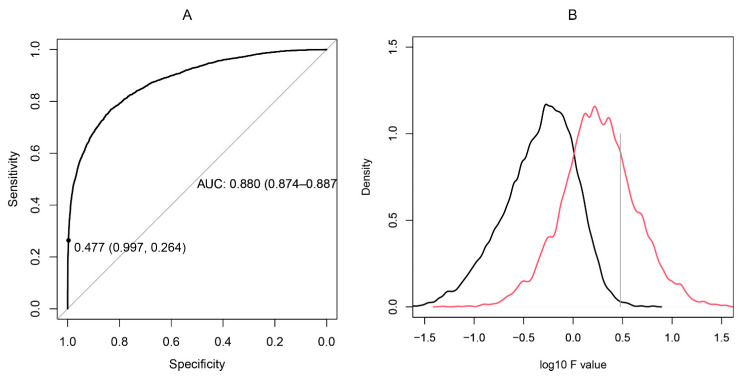
(**A**): ROC curve constructed on the basis of the F statistic of 4150 pairs of overlapping neighboring peptides from the studied proteins compared to a set of 10,000 pairs of dissimilar peptides selected to have a longest common subsequence of fewer than three residues. (**B**): Distributions of the logarithms of F statistic for dissimilar sequences (black) vs. overlapping sequences (red). The optimal tradeoff between sensitivity and specificity was found for F = 1 (1-specificity = 0.833, sensitivity = 0.769), but the F level of 3 was selected as a threshold (1-specificity = 0.977, sensitivity = 0.264) selecting the upper quartile of most cross-reactive pairs only.

The reactivity profiles of a pair of peptides with a set of N sera may be represented as a comparison between N groups, each of two points. The F statistic is the ratio between (a) the variance of the means of the groups and (b) the mean of the variances within the groups. Thus, when two profiles are similar, the two points in each group are close while the overall reactivity differs much more between groups (Figure 2). To measure the capacity of the F statistic to differentiate between cross-reactive and non-cross-reactive peptides, the set of maximally overlapping pairs of peptides was compared to a set of peptide pairs containing unrelated sequences (Figure 3). The F statistic correctly classified the two types of peptide pairs with an area under the ROC curve of 0.88. The optimal F value suggested by the ROC curve yielded excessive cross-reactivities, including 20% false positive pairs. To reduce the complexity of the system of cross-reactivities and to increase the specificity of the criterion, the critical value of F was selected to be 3. In this way, we could construct a graph of peptides as vertices connected by edges wherever the respective pair had an F statistic with the studied set of sera greater than 3. Thus, some peptide pairs with low but significant cross-reactivity were left out to concentrate only on the most prominent relations. The values of F > 3 were kept as weights of the edges of the graph.

#### 2.2.2. Overview of the Properties of the Reactivity Graph

**Figure 4:**
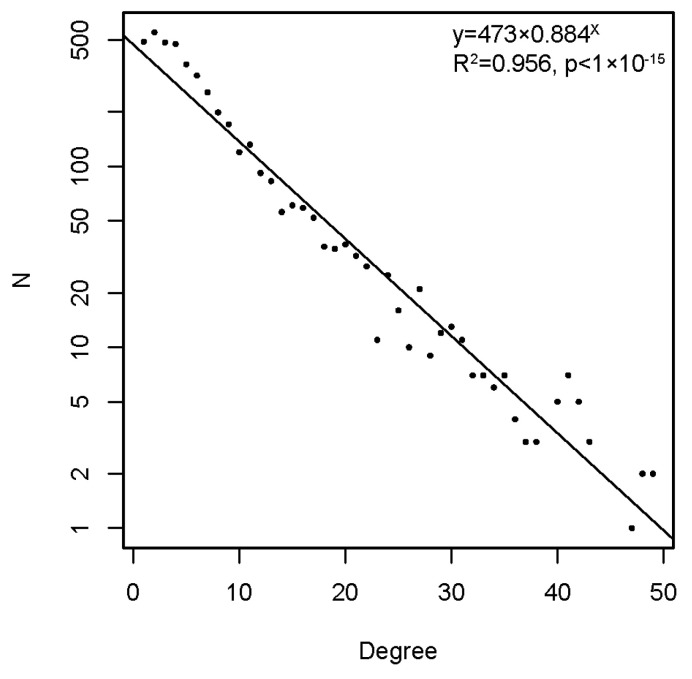
The degree distribution in the reactivity graph follows an exponential low.

**Figure 5:**
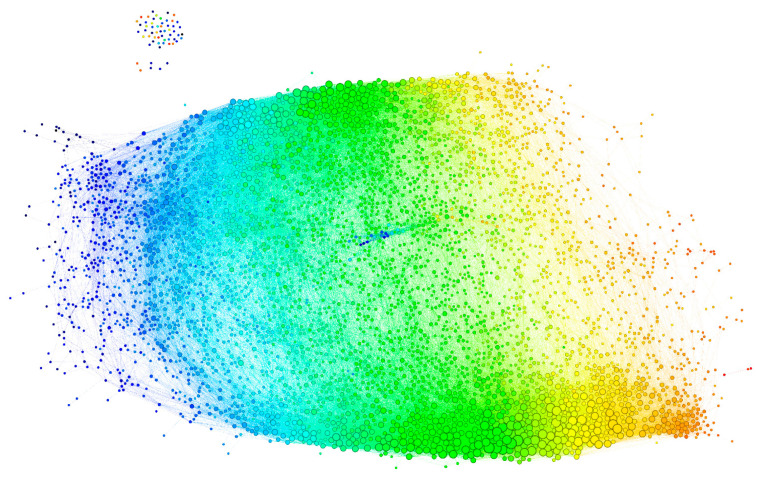
Visualization of the reactivity graph. The Force Atlas 2 algorithm from Gephi software was used. The vertices are colored according to the mean intensity of the IgM binding (from blue for the lowest through green to orange for the highest). The edges inherit the color of the adjacent vertices. The size of the vertices is proportional to their degree. The small group of vertices in the top left corner represents the singlets and the disconnected pairs.

The graph, thus constructed, had 4512 vertices and 77,914 edges. The diameter of the graph—the shortest path between the farthest vertices without edge weights, was 12. The graph consisted of a large connected component of 4455 peptides and 52 disconnected peptides as well as three pairs unrelated to the rest. The vertex degree (number of neighbors) had an exponential distribution, which is a property of networks evolving by random attachment (Figure 4) [28]. Table 1 contains the ranking of the proteins and epitopes studied by modularity (clustering of vertices with more edges within the clusters than between the clusters) in the reactivity graph. It shows the relatedness of the reactivity profiles of the linear epitopes in each protein. The collection of diverse viral epitopes as a group was quite separate from the self-epitopes as a class of reactivities. Overall, a highly connected graph of IgM cross-reactivity was obtained with a set of 4512 peptides and 21 individual sera. The visualization of the graph was calculated using the Force Atlas 2 algorithm in Gephi software (Figure 5) [29].

#### 2.2.3. Visualization of the Reactivity Graph

**Figure 6:**
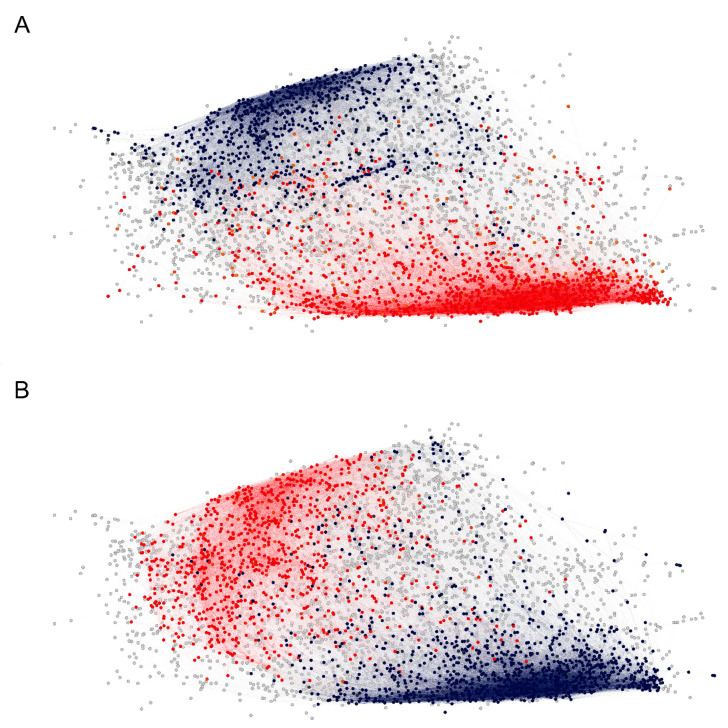
The two dense clusters of reactivity profiles appear to correlate with ABO blood group antigen expression, (**A**) vertices for which the neighborhood-averaged profiles show higher (red) or lower (blue) mean reactivity in patients carrying blood group antigen A. (**B**) The same as (**A**) for patients carrying blood group antigen B.

This visualization is a provisional embedding for illustrative purpose, so the dimensions are not intended to be interpretable. Nevertheless, the form and the relationship between the clusters of vertices are interpretable. The long horizontal axis along which the vertices are spread correlated with the mean intensity of the IgM reactivity in the profiles (Figure 5—color code). The high-degree vertices (with many neighbors) are concentrated mostly in two large clusters parallel to this axis (Figure 5—size code). The spread in the vertical direction of the graph layout seemed to separate two dense clusters of profiles, which differed reciprocally in the mean expression of reactivities to the blood group A and B antigens (Figure 6), but the nested ANOVA test over the vertex neighborhoods failed to find significant differences. The individual proteins mapped to diverse portions of the graph with the larger ones (e.g., Erb-B2) covering all regions (Supplementary Figure S2). Thus, the features of the reactivity graph related to the local structural and chemical properties of the sequences defining antibody binding rather than overall antigen specificity. IgM (poly)autoreactivity did not seem to reflect antigen-driven expansion, but the overall network topology of random attachment suggested antigen-driven selection.

#### 2.2.4. Topology of the Reactivity Graph

**Figure 7:**
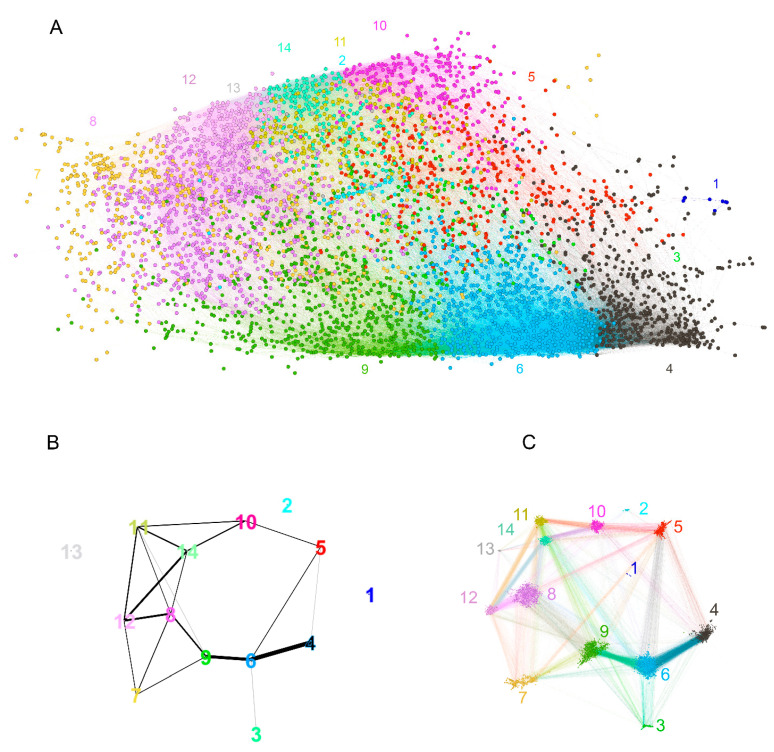
Reactivity graph topology. (**A**) Color-coded labeling of the clusters defined by recursive Louvain clustering. The numbers indicate the number of the cluster and are of the cluster’s color. (**B**) Graph of the clusters with edges based on thresholded modularity of the two adjacent clusters (the modularity was calculated on the induced subgraph containing only the two clusters considered). The colors of the clusters are the same as in (**A**). The threshold used for the plot was selected to illustrate the dominant persistent homology (cycle 5, 6, 9, 8, 14, 10). This proves that the graph has relatively sparse connectivity in the region between those clusters, which is impossible to visualize in the 2D layout (**A**) because they are overlayed. (**C**) Rearrangement of the graph to show the respective topology preserving all edges. The numbers are the numbers of the clusters, and the color code is the same as that in (**A**,**B**).

The layout of the graph was constrained to two dimensions, and in view of its high connectivity, significant topological information could be lost. Indeed, a hierarchical recursive Louvain clustering and subsequent optimal tree cutting yielded 15 clusters (communities) with higher edge density within than between the clusters (Figure 7A). Fourteen of the 15 clusters were in the large component of the graph, and the fifteenth represented the disconnected peptides. The color-coded representation of the clusters showed a significant overlap of distinct clusters in the middle of the layout used. To better define the topology, the pairwise modularity measure between each two clusters was used as a dissimilarity measure to construct a weighted graph of the 14 connected clusters (Figure 7B,C). Following the topological data analysis (TDA) concept of filtration, by changing the threshold of the modularity, a series of graphs was produced, which were examined for the occurrence or disappearance of cycles—closed paths [30]. A large cycle was observed to appear early (at low modularity) and disappear late (at high modularity), corresponding, in topological context, to a persistent homology. Simplifying the TDA approach, a large “hole” in the graph topology was observed, which was not evident from the layout used. Arranged around it were the clusters 5, 6, 8, 9, 11, 14, and 10 (Figure 7B). Another layer of the topology was formed by cluster 7, which spanned the whole layout being connected mostly to clusters 8, 9, and 12. It is important to note that the notion of (dis)connectedness in this TDA approach was relative, meaning that it depended on a threshold of the modularity between the clusters. Thus, cluster 7 was connected weakly all the way to clusters 6 and 5 and even with a couple of edges to 4 (Figure 7C).

**Table 1:** Epitopic diversity measured by modularity of the reactivity graph. Each protein is isolated as a cluster of vertices compared to the rest of the graph. The modularity of this clustering is then compared to a bootstrapped distribution of the modularity of the same number of random vertices in this graph to calculate the z-score. The latter indicates the actual magnitude of the compactness of the cluster, meaning how closely related are the reactivity profiles of the linear epitopes of this protein. Although diverse, the collection of all viral epitopes studied showed high modularity compared to the self-antigen epitopes. Louvain clustering refers to the clustering of the whole graph in 15 clusters (Figure 7A). The column “Viral” indicates the viral proteins and epitopes by shading.

Graph Clustering Based On	Modularity	Z-score	Mean Degree	Viral
Louvain Clustering	5.72 × 10^−1^	514.683	34.536	
LANA Human herpesvirus 8	1.75 × 10^−3^	43.096	34.462	
Cancer/testis antigen 1 (NY-ESO-1) (UniProt P78358)	5.42 × 10^−3^	38.299	35.247	
Histone H1.2 (UniProt P16403)	5.14 × 10^−3^	34.129	23.245	
Histone H4 (UniProt P62805)	1.84 × 10^−3^	28.544	43.268	
All Viral	2.20 × 10^−2^	27.886	34.172	
Protein SSX2 (Cancer/testis antigen 5.2) (UniProt Q16385)	3.31 × 10^−3^	24.357	56.138	
L-dopachrome tautomerase (UniProt P40126)	7.87 × 10^−3^	20.367	32.817	
envelope glycoprotein Human T-lymphotropic virus 1	4.24 × 10^−3^	20.358	44.568	
Carcinoembryonic antigen-related cell adhesion molecule 5 (UniProt P06731)	9.30 × 10^−3^	18.928	31.683	
G2/mitotic-specific cyclin-B1 (UniProt P14635)	5.95 × 10^−3^	18.584	32.641	
Melanocyte protein PMEL (UniProt P40967)	7.40 × 10^−3^	15.590	36.761	
Epstein-Barr nuclear antigen 1 Human herpesvirus 4	1.04 × 10^−3^	14.264	27.200	
Transcription factor SOX-2 (UniProt P48431)	3.23 × 10^−3^	13.371	42.364	
Cellular tumor antigen p53 (UniProt Q2XN98)	4.12 × 10^−3^	13.130	33.090	
Mammaglobin-1 (UniProt Q13296)	8.21 × 10^−3^	12.978	17.385	
Stromelysin-3 (MMP11) (UniProt P24347)	4.66 × 10^−3^	12.825	34.304	
Myc proto-oncogene protein (UniProt P01106)	3.92 × 10^−3^	12.220	36.788	
HLA class I histocompatibility antigen A-36 alpha chain (UniProt P30455)	3.23 × 10^−3^	12.127	32.730	
Receptor tyrosine-protein kinase erbB-2 (UniProt P04626)	1.05 × 10^−2^	12.043	32.769	
E7 protein Human papillomavirus type 16	7.10 × 10^−4^	11.119	27.237	
L2 protein Human papillomavirus type 16	2.00 × 10^−4^	11.004	19.667	
Probable protein E4 Human papillomavirus type 16	3.40 × 10^−4^	10.493	54.455	
BZLF1 Human herpesvirus 4	1.34 × 10^−3^	10.168	31.873	
Putative HTLV-1-related endogenous sequence (p25) Homo sapiens	2.37 × 10^−5^	9.643	4.000	
Tyrosinase (UniProt P14679)	3.31 × 10^−3^	8.536	34.015	
Replication protein E1 Human papillomavirus type 16	6.30 × 10^−5^	8.226	101.667	
Myelin-oligodendrocyte glycoprotein (UniProt Q16653)	1.38 × 10^−3^	7.624	29.293	
Claudin-6 (UniProt P56747)	1.25 × 10^−3^	7.552	33.951	
Capsid protein VP26 Human herpesvirus 4 (strain B95-8)	1.21 × 10^−4^	7.409	20.091	
Prostate-specific antigen (UniProt P07288)	1.37 × 10^−3^	6.925	34.821	
envelope glycoprotein Human T-lymphotropic virus 2	1.15 × 10^−4^	6.688	45.455	
Ricin precursor Ricinus communis	2.22 × 10^−4^	5.372	22.964	
L1 protein Human papillomavirus type 16	4.81 × 10^−4^	5.340	30.340	
Carbonic anhydrase 1 Homo sapiens	1.83 × 10^−4^	5.096	41.682	
tax protein Human T-lymphotropic virus 1	7.98 × 10^−5^	4.650	48.000	
Pr gag-pro-pol Human T-lymphotropic virus 1	1.92 × 10^−4^	4.337	24.933	
small viral capsid antigen Human herpesvirus 8	1.31 × 10^−4^	4.197	32.524	
TCR gamma alternate reading frame protein (UniProt A2JGV3)	1.23 × 10^−4^	4.114	34.727	
L1 Human papillomavirus type 33	2.39 × 10^−5^	3.795	46.250	
E7 protein Human papillomavirus type 18	2.27 × 10^−5^	2.807	45.667	
Protein X Hepatitis B virus	3.51 × 10^−5^	2.052	28.000	
major capsid protein Human papillomavirus type 6	1.43 × 10^−5^	1.921	51.000	
E2 protein Human papillomavirus type 16	2.37 × 10^−5^	1.801	19.222	
ribonucleoside-diphosphate reductase large chain Human herpesvirus 4	1.60 × 10^−5^	1.650	30.500	
Plasminogen-binding protein pgbA Helicobacter pylori	−1.39 × 10^−8^	0.353	13.000	
hippocampal 38K autoantigen protein Homo sapiens	−7.91 × 10^−8^	0.163	15.500	
Early antigen protein R Human herpesvirus 4 (strain B95-8)	−1.06 × 10^−7^	0.155	12.667	
E2 Human papillomavirus type 18	−1.23 × 10^−7^	0.100	21.500	
Hbx protein Hepatitis B virus	−1.88 × 10^−7^	0.076	25.500	
Latent membrane protein 2 Human herpesvirus 4 (strain B95-8)	−1.44 × 10^−7^	0.051	14.667	
L2 Human papillomavirus type 6	−5.81 × 10^−7^	0.040	23.000	
E2 protein Human papillomavirus type 6	−3.96 × 10^−7^	0.035	36.500	
rex protein Human T-lymphotropic virus 1	−3.03 × 10^−6^	−0.008	21.100	
T-cell receptor beta chain; TCR Homo sapiens	−5.53 × 10^−7^	−0.020	43.000	
E6 protein Human papillomavirus type 16	−2.41 × 10^−6^	−0.034	18.400	
K8.1 Human herpesvirus 8	−3.18 × 10^−6^	−0.183	29.857	

**Table 2:** Tumor antigens included in the microarray and the number of peptides. The antigens are ordered in an ascending order relative to the mean IgM reactivity.

Protein		N
Mammaglobin-1 (UniProt Q13296)	SCGB2A2	39
T-cell receptor beta chain; TCR Homo sapiens	TCRb	2
Carbonic anhydrase 1 Homo sapiens	CAH1	21
TCR gamma alternate reading frame protein (UniProt A2JGV3)	TCRg_alt	22
Tyrosinase (UniProt P14679)	TYRO	267
Myc proto-oncogene protein (UniProt P01106)	Myc	212
Prostate-specific antigen (UniProt P07288)	PSA	123
Carcinoembryonic antigen-related cell adhesion molecule 5 (UniProt P06731)	CEA	325
Protein SSX2 (Cancer/testis antigen 5.2) (UniProt Q16385)	SSX*2*	87
Myelin-oligodendrocyte glycoprotein (UniProt Q16653)	MOG	116
Transcription factor SOX-2 (UniProt P48431)	SOX2	151
Claudin-6 (UniProt P56747)	CLDN6	103
Receptor tyrosine-protein kinase erbB-2 (UniProt P04626)	erbB2	620
L-dopachrome tautomerase (UniProt P40126)	DCT	252
HLA class I histocompatibility antigen A-36 alpha chain (UniProt P30455)	HLA_A36	178
Stromelysin-3 (UniProt P24347)	MMP11	237
Melanocyte protein PMEL (UniProt P40967)	PMEL	323
Cellular tumor antigen p53 (UniProt Q2XN98)	p53	200
Histone H1.2 (UniProt P16403)	H1_2	98
Hippocampal 38K autoantigen protein Homo sapiens	ELAVL2	2
Cancer/testis antigen 1 (NY-ESO-1) (UniProt P78358)	NY-ESO-1	89
G2/mitotic-specific cyclin-B1 (UniProt P14635)	CCNB1	209
Histone H4 (UniProt P62805)	H4	41
Putative HTLV-1-related endogenous sequence (p25) Homo sapiens	HRES1	2

The highly connected graph contained 239,349 maximal cliques of 3 to 38 vertices (Supplementary Figure S3). A clique is a complete subgraph, one in which all vertices are connected. A maximal clique is a clique that is not a subgraph of another clique but maximal cliques can overlap with a portion of their vertices. The cliques, as well as neighborhoods, in the reactivity graph can be interpreted as images of individual specificities—especially maximal cliques since each represents a group of peptides that are all cross-reactive. From here on for simplicity by cliques, we mean maximal cliques of more than two vertices. The cliques were of widely varying sizes with the large cliques formed in the dense clusters overlapping extensively. The 3 and 4 cliques contained 97.3% of the 4263 vertices in the large component, and the cliques with up to 10 vertices covered the whole graph. The larger cliques were formed by recombining vertices participating in smaller cliques and formed the densest regions of the graph. Only 424 peptide reactivities formed 82,334 cliques of more than 20 vertices.

#### 2.2.5. Mapping Sequence and Specificity Data onto the Graph Topology

A number of motifs were extracted from the set of peptides found in the cliques of 20 sequences and larger using the XSTREME algorithm from the MEME suit (https://meme-suite.org/, accessed on 13 December 2022 [31]), and they corresponded to known profiles found mostly in bacterial antigens (Supplementary File S2). Large cliques of cross-reactive linear epitopes can represent highly similar sequences and/or abundant reactivities, e.g., from highly concentrated public antibody clones, which would have a wider range of cross-reactivities above a threshold of biologically relevant binding even at lower affinity. Entropy comparisons between the sets of sequences in the small (<5) vs. the large (>20) cliques showed no significant difference (linear models, *p* < 0.05). The k-mer distance between the sequences showed an increase of only 1% in similar sequences in the large cliques (Chi square test, *p* < 0.05). Therefore, it was hypothesized that the large cliques are not due to degenerate epitopes but most probably to abundant oligoclonal public reactivities.

**Figure 8:**
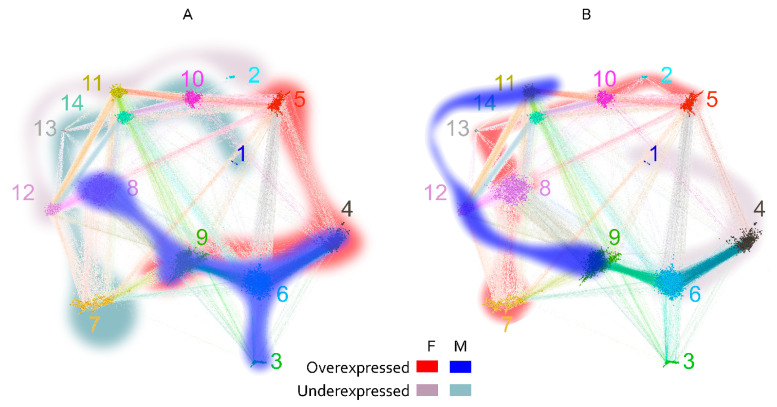
Map of significantly different cross-reactivity to the major clusters between blood group A (**A**) and B (**B**) antigen-carrying patients separately for men (blue scale) and women (red scale). There is a greater overlap between the sexes in the cross-reactivity spectrum in blood group A (clusters 4 and 9) than in blood group B. Some clusters have high cross-reactivity related to the presence of both A and B antigens (5, 8, and 9). Both factors define to a great extent the major patterns of cross-reactivity encoded in the graph topology. The two high-density regions containing the large cliques are also at the center of this dichotomous cross-reactivity—clusters 4, 6, and 9 for blood group A and clusters 11, 12, 13, and 14 for blood group B.

The coincidence of the clusters of high density containing the large cliques and the parts of the graph with apparently divergent anti-A and anti-B reactivity (Figure 6) suggested that major anticarbohydrate reactivities such as blood group or, for instance, anti- alpha Gal could be in part an explanation for the existence of this cluster of broad cross-reactivity. Indeed, the difference of the reactivity to these clusters in patients carrying blood group A or B antigens proved to be significant at the level of the graph clusters. Based on the topological map of the graph (Figure 7), a significant difference between blood group antigen A- and B-carrying patients in the reactivity to many clusters was observed (Figure 8). The pattern was different for men and women, especially in the case of blood group B. Another aspect of the IgM IgOme is the frequent cross-reactivity with immunoglobulin J region linear epitopes, which amounts to idiotypic interactions [8,27]. In the current experimental setting, again, this was readily demonstrable. The studied peptides contained 157 unique 7-mers, which can be found in human immunoglobulin J regions, and 12% (546/4512) of the peptides contained at least one 7-mer idiotope and 194 contained from 2 to 11 idiotope 7-mer frames. The content of idiotopes in the cliques increased with the size of the cliques, although idiotopes could be found in all size of cliques (Supplementary Figure S4).

The dichotomous separation of cross-reactivities associated with blood group antigen expression covered a wide and diverse set of peptides and was proven to be among the major factors defining the topology of the graph being related to dense clusters and large cliques.

**Figure 9:**
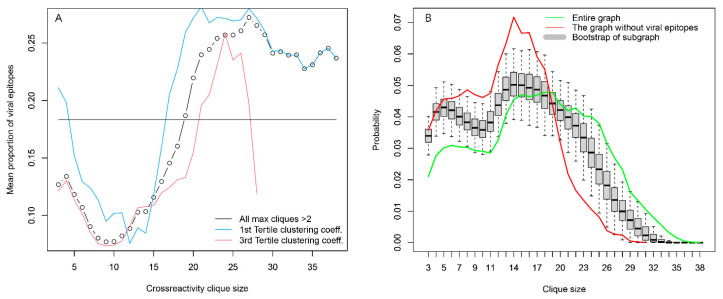
Viral epitopes are an important organizing element of the IgOme. (**A**) Distribution of viral epitopes as a function of clique size. They are very common in the overlapping large cliques without being over-represented in the set of peptides that are found in large cliques. This suggests that they are central elements on which many large cliques overlap. Viral epitopes are also found mostly in cliques with a lower capacity to classify the tumor patients (blue and red lines). (**B**) Viral epitope (probably preimmune) reactivities are found in many large cliques of the reactivity graph because they are most often the ones in which the large cliques overlap. Omitting them leads to a graph with significantly fewer large cliques (red line) compared to the bootstrapped-induced subgraphs lacking the same number of randomly sampled vertices.

Another possible source of highly expressed public IgM reactivities, testable by the set of antigens used, was antiviral responses, although this would be more relevant in the case of IgG and IgA antibodies. More viral linear epitopes than self-epitopes participated in the large cliques (>20 vertices), and the opposite was true for the small cliques (<5 vertices) (Supplementary Figure S5). This effect was stronger for patients carrying blood group A antigen. The proportion of viral epitopes in the peptides found in large cliques was the same as for the whole graph, but the proportion of viral epitopes in the large cliques was 2.5 times higher than that in small ones (Figure 9A). This could be evidence that the viral epitopes represent most often the vertices in which the large number of large cliques overlap, in other words—they have more cross-reactivities than the self-antigens and may represent a substantial part of the central reactivities around which the large cliques organize. Indeed, an induced subgraph of the reactivity graph lacking the viral epitopes had a clique size distribution significantly shifted to smaller sizes as compared to the bootstrapped distribution of such induced subgraphs lacking the same number of randomly sampled vertices (Figure 9B).

Interestingly, the most prominent viral specificities were several HTLV1 epitopes (Supplementary Figure S6). The latter is probably cross reactivity with unknown specificities since the patients are from the Bulgarian population, which has almost no exposure to HTLV1 as shown by regular screening of donor blood ([32] and according to Prof. Rada Argirova, personal communication). Therefore, the high reactivities to viral sequences are probably a phenomenon of non-self-recognition rather than immunological memory which would not be typical of IgM.

Altogether, these data indicate that the large clusters of the highly connected parts of the graph likely reflect the cross-reactivity of highly concentrated public anticarbohydrate antibodies and non-self-recognition.

### 2.3. Reactivity Graph and the IgM Repertoire Changes in Brain Tumors

**Figure 10:**
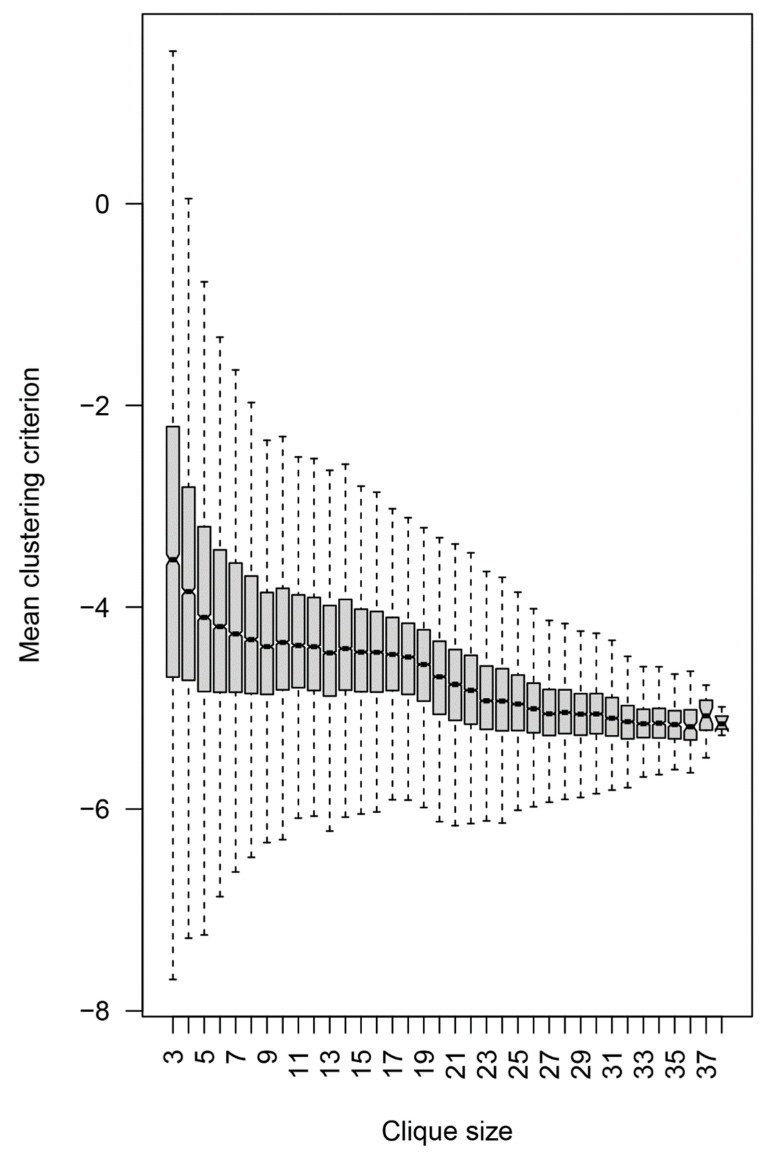
Dependence of the mean clustering criterion on the clique size. The composite clustering criterion based on the sum of the Dunn’s and the Baker–Hubert gamma criteria, and connectivity was used to measure the separation of the point clouds corresponding to the three diagnostic groups. Up to 100 samples of three vertices (peptides) from each clique with more than three vertices were used. The reactivity values of the 21 patients in each of these samples were used to calculate the criterion, and the values for each clique were averaged. Thus, all values were based on three-dimensional spaces. Without feature selection, the separation between the diagnostic groups was poor, hence the negative values, but the extreme values of the criterion were positive for the 3- and 4-cliques.

To focus on the specificities relevant to potential brain tumor classifiers, the peptide reactivities in the individual cliques were further tested for their capacity to separate the three diagnostic groups. The diagnoses were mapped onto the reactivity data for each 3-clique or the mean of all combinations of three peptides in the larger cliques. Sampling of the cliques larger than three vertices was done to compare the separation at the same dimensionality (Figure 10). A composite clustering criterion was used to measure the separation between the groups (see Section 2) [8]. The comparison showed that the capacity of the sets of peptide reactivities to classify the diagnostic groups depended inversely on the size of the cliques the peptides were found in. Thus, the IgM reactivity profiles correlating with brain tumors favored rare self-reactivities.

**Figure 11:**
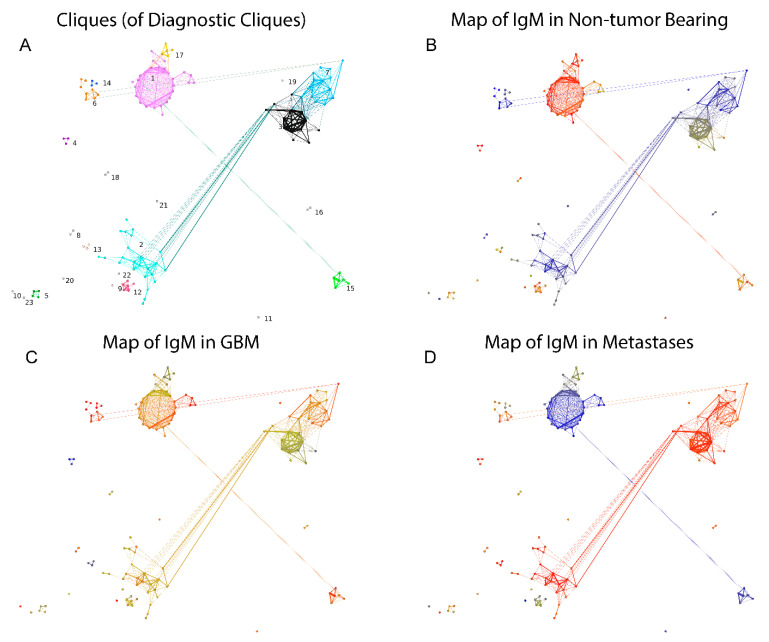
The graph of DClq (GpCl). Each vertex is a clique in the reactivity graph, and the edges correspond to the existence of common vertices between the connected cliques with the size of the overlap encoded in the edge weights. The layout follows roughly the map of the reactivity graph. (**A**) Louvain clustering yielded 23 clusters of GpCl. (**B**) Map of the reactivities over (red)/under (blue) expressed in non-tumor bearing patients. (**C**) Map of the reactivities over (red)/under (blue) expressed in GBM patients. (**D**) Map of the reactivities over (red)/under (blue) expressed in brain metastasis patients.

The potential correlation of some IgM reactivities with the presence of tumors was further explored by focusing on the candidate peptides thus identified. To this end, the clustering criterion calculated on the reactivity data for each clique of size between 3 and 10 was compared to a bootstrapped 99th percentile of the clustering criterion on the reactivity data for 10^4^ random samples of peptides of the same size as the tested clique. The selected 130 potentially diagnostic cliques (DClq) of reactivities were of sizes 3 to 7 (79/1.57% of the 3 cliques, 26/0.4% of the 4 cliques, 12/0.17% of the 5 cliques, 11/0.15% of the 6 cliques and 2/0.03% of the 7 cliques). These represented 228 peptides. The predominant motifs extracted from these peptides using the MEME suit of algorithms are listed in Supplemental File S3. To better understand the relationship between DClq, each one was represented as a single vertex in a new graph—graph of DClq (Gp(ositive)Clq). The edges in the GpClq were between cliques that overlapped, and the number of common vertices was used as a weight for the edges. GpClq had 18 components, 14 of which had fewer than four vertices. The vertices were further partitioned in 23 clusters using Louvain clustering (Figure 11A). Interestingly, the connected components of GpClq consisted mostly of large cliques (cliques of cliques). These are probably groups of cross-reactivities, which would probably turn into even larger cliques in the original graph at a different threshold of joining. Mapping the mean reactivity by diagnostic group onto the GpClq showed a significant difference between many of the cliques, which was expected since this subgraph of the reactivity graph was selected based on its capacity to differentiate the diagnostic groups (Figure 11B–D). Reactivities in the GpCLq cliques 1, 15, and 17 were prominently lost in one or both of the tumor-bearing groups. They all consisted exclusively of self-antigen epitopes. As shown previously [8,27], IgM reactivity is often lost in pathology rather than gained.

The antigens significantly overrepresented in the GpClq cliques included 20/24 self-antigens and 8/29 non-self-antigens including the linker used in the microarray (GSGSGS). The latter was meant to be an inert flexible linker used as a separator between the protein sequences by the peptide manufacturer. Unexpectedly, it showed not only very high reactivity with human IgM but also involvement in cancer-induced IgM changes and is the most prominent motif in the set of DClq (Supplementary File S3). Apart from its flexibility that facilitates promiscuous-induced fit recognition of the adjacent short sequences, it is interesting to note that this sequence is found in immunoglobulin J genes and represents an idiotope. Actually, 97/546 hypothetical idiotope-containing sequences had this motif. It is also interesting to note that this motif was the most significant hit in the large clique set of peptides (Supplementary File S2) and was identified as a V8 family serine protease and beta lactamase sequence profile. Overall, three of the cliques of GpClq concentrated a disproportionate number of potential idiotopes—cliques 6, 10, and 14. These cliques were also associate with increased reactivity of their peptides in brain tumors (mostly GBM, Supplementary File S1).

Thus, the diagnostically relevant reactivities included disproportionately more self-antigens. The viral epitopes found in the cliques were from EBV (EBNA and BZLF1) and HPV16 (E6 and E7), with one from human herpesvirus 8 LANA and one from HTLV1 Env. The most represented self-antigens were (in this order) Myc, G2/mitotic-specific cyclin-B1 (CCNB1), tyrosinase (TYRO), ErbB2, PSA, stromelysin (MMP11), and p53. These associations reached statistical significance for the following antigens and cliques: CCNB1 (1, 3, 7), Myc (12, 17), TYRO (12, 20), H4 (1, 19), PSA (8, 15), ErbB2 (23), SSX2 (9), CEA (9), and the viral epitopes from EBV (2, 11, 18), HPV (21, 22), and HHV8/KSV (2) (Supplementary File S1). The contribution of the different antigens to the reactivities in GpClq by clique is visualized by PCA and biplots in Supplementary Figure S7A–D. It is important to emphasize that this proof-of-principle study is probably limited in its capacity to extract comprehensive information on particular TAA reactivities due to the small number of patients included and the high variability of clinical data.

Interestingly, the HTLV1 reactivity segregated from the widespread herpes and papilloma virus reactivities and with the self-antigens along the 6th component. This strengthened the hypothesis that some cross-reactivity with self may explain its central role while the virus is unknown to the population studied.

Finding that reactivity graph clique sorting by size provides a suitable correlate for potential tumor-associated reactivities suggested a tool for filtering the IgM IgOme features to be used in a subsequent machine learning model.

### 2.4. Proof-of-Principle Classifier

**Figure 12:**
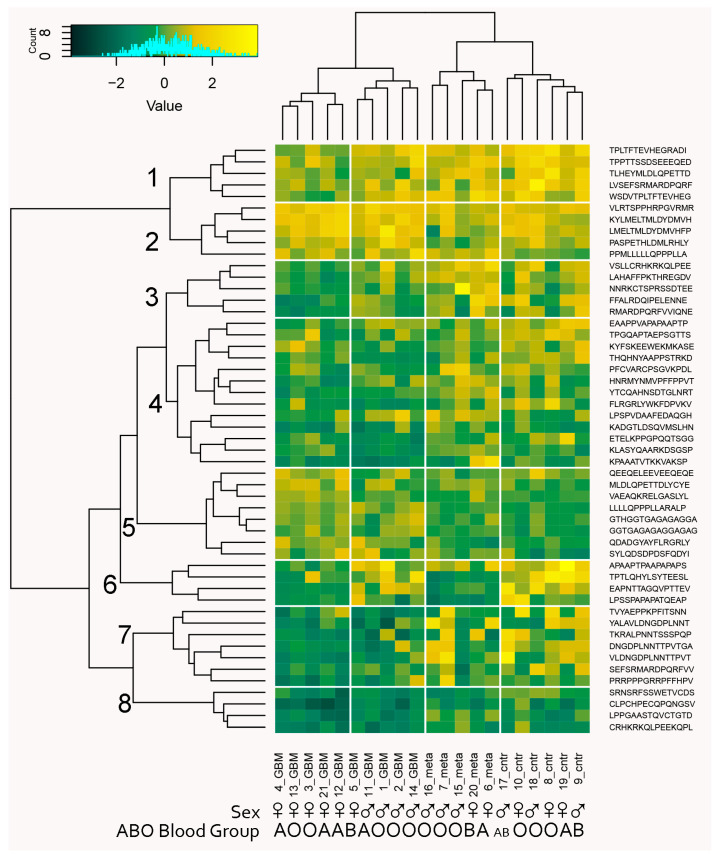
Biclustering heatmap plot of the patients’ IgM reactivities with the classifier profile of 51 peptides separating the three diagnostic groups. The profile provides a good clustering of both patients by diagnostic group and of peptides that fall into eight clusters. Only cluster 5 contains reactivities that were significantly increased in tumor-bearing patients, while the reactivities in clusters 1, 4, 6, 7, and 8 tended be lost in one or both of the tumor-bearing patient groups. Interestingly, clusters 1, 3, 6, and 7 distinguished two groups in the GBM group, which correlate with sex while sex was not used as a parameter in the feature selection.

High-throughput IgM binding data can be utilized as a source of biomarkers representing subsets of peptide reactivities (immunosignatures [33]) that collectively probe relevant IgM polyspecificities. A key problem is the filtering of the large number of peptides before searching extensively for high-performing subsets using recursive feature elimination [8]. The filtering step in feature selection ensures focusing on a limited set of meaningful reactivities (e.g., 10^2^–10^3^ peptides) out of a collection of n × 10^3^ peptides. This is necessary because of the astronomical number of possible combinations of features in the ultimate classifier of suitable size [8]. The generalization a reactivity graph offers might help in feature selection as has been shown previously [34,35]. A major distinction between the different parts of the reactivity graph was the density. Interestingly, the peptides participating in different size cliques proved to have different capacities to differentiate the three diagnostic groups (Figure 10). Therefore, filtering the peptides by extracting them from cliques that were better in separating the diagnostic groups could provide the necessary filtering step for an optimal machine learning classifier. The filtering step applied selected the 3- and 4- cliques that separated the diagnostic groups. The 95th percentile of the distribution of the criterion in random sequence samples of the same size as the tested clique was used as a cut off. The obtained reduced set of peptides was ultimately subjected to recursive feature elimination [8] to derive a (locally) optimal feature set. The capacity of this algorithm to produce efficient classifiers was tested by wrapping the whole procedure (from graph generation and clique definition through filtering to recursive elimination feature selection) in a leave-one-out cross validation. Thus, 21 sets of features (peptides) were generated. Each was derived from a separate reactivity graph based on 20/21 of the patient and contained in the average 42 peptides. Each feature set was next tested for its capacity to predict correctly the patient excluded. The reactivity data from all patients with each of these sets of peptides were first mapped onto two dimensions using multidimensional scaling followed by generating a support vector machine model (Supplementary Figure S8). Surprisingly, this cross validation yielded 100% correct predictions. This was much better than our previous classifier based on the reactivity of individual mimotope sequences [8]. To produce the ultimate predictor set, the 21 sets of peptides were compared, and those that were found in more than four of the sets were retained. The threshold for this decision was selected based on the clustering criterion for the maps of the diagnostic groups with each set of features. This yielded 51 peptides that classified correctly the three groups of patients (Figure 12).

The 51 peptides’ reactivities clustered in eight profile clusters (PrCl), five of which (1, 4, 6–8) showed a significant mean reduction in reactivity in the tumor groups compared to the control group, while only one (5) showed a significant increase in the GBM group (ANOVA, *p* < 0.05). Thirteen of the 24 self-antigens were represented, and none showed a correlation with a particular PrCl. On the other hand, the viral antigen epitopes (HHV8/KSV LANA, HPV16 E7, and EBV nuclear antigen) were found mostly in PrCl 5 with increased reactivity in brain tumors, especially in metastases (Supplementary File S1). With respect to blood group cross reactivities, the brain tumor immunosignature peptides in PrCl 3, 6, and 7 showed a higher intensity in blood group B-expressing patients compared with those in PrCl 1—lower in blood group A-expressing patients. Reactivities overexpressed in men were also found in PrCl 7 (Supplementary File S1).

## 3. Discussion

In the present study, the patterns of reactivity of IgM with peptides from known TAA or cancer-related linear B cell epitopes were studied in brain tumor patients. A great diversity of patterns of reactivity in the individual cases was observed. It is conceivable that the immune reorganization due to the presence of a malignant tumor was significant, but the number of altered reactivities was in the hundreds and spanned most of the tested TAA. The changes were mostly epitope specific and not antigen specific. The major mechanism of changes in the IgM repertoire may be fluctuations caused by inflammatory signals, fixation of the antibodies in the tissues and immune complexes or changes in the composition of the repertoire of the respective plasma cells in the bone marrow and only partially the classical immune responses (primary or T-independent). These characteristics combined with different accessibility of tissue antigens under different conditions may explain the number of reactivities found in non-tumor bearing patients and lost in those with tumors. This is also in line with previous findings of numerous IgM reactivities involved in tumor surveillance and tumor diagnosis. Disappearing IgM reactivities is an interesting recurring observation of the nature of the changes in the IgM repertoire in pathology [8,27]. It may be related to the constitutive expression, especially of natural antibodies possibly combined with increased catabolism when the target antigens’ concentration/accessibility is increased. It is possible that the normal antigenic landscape is in equilibrium with the steady state production of natural antibodies, but the increase in the abundance of some overexpressed or non-physiologically exposed antigens in tumors may tilt this balance (temporarily) to lower reactivity of the respective IgM reactivities. It is possible also that specificities might get lost due to the activation of B2 or memory B cells, which then die (due to overstimulation) [36] or differentiate into IgG producing cells.

The IgM repertoire consists to a great extent of inherently polyspecific clones, and most of the bound structures are expected to be conformational epitopes. These two facts should be reflected in the reactivities to the 15-me epitopes of known TAA tested. While some of the observed reactivities may be actual autoreactivities to accessible epitopes involved in tumor surveillance, others may be simply cross-reactivities apparent only in vitro with little significance in vivo.

Physiologically autoreactive antibodies could tag native tumor-associated antigens (TAA) simply as a result of overexpression or ectopic presence. Quantitative self-tolerance is comprehensible mostly in terms of physiologically maintained moderate to low titers of physiologically autoreactive antibodies [24]. Therefore, it is not surprising that the nAAb compartment’s involvement in the immune surveillance has been demonstrated [23,25,37,38,39] although it is known to be of limited inducibility mostly by danger signals rather than by specific stimulation [40,41].

There is a sufficient body of evidence for positive selection of the B cell repertoire on self-antigens [42,43,44,45,46]. This could explain the close relationship of the IgM repertoire with self-antigens without the induction of some form of immune response. The random attachment cross-reactivity graph is evidence of the IgM repertoire evolution by recruitment of clones related in cross-reactivity to the already available ones. The reason for this could be the selection by a relatively constant antigenic landscape. Finding this property of the IgM cross-reactivity graph comes as a confirmation of previous data on the mimotope IgOme graph [27] as well as from a graph of repertoire sequencing data that showed that this property is characteristic only of the preimmune IgM repertoire [3].

Rarely has IgM reactivity been used to extract diagnostic reactivity profiles. In mice, IgM reactivity to a set of autoantigens provided more consistent tumor-related patterns than IgG [11,38,39]. Stafford et al. (2016) demonstrated that IgM immunosigantures to random peptides were more consistent than IgG, both inter individually and in time, which was interpreted in favor of IgG as a richer source of information [9]. The opposite argument stresses the necessity of antibody repertoire profiles to be sufficiently consistent and relatively independent of the individual’s immunological history in order for them to be sources of generalizing classifiers or predictors of phenomena in the internal environment. Even just for IgM, we observed such a diversity of IgM reactivity that sets of patients’ sera, which differed by only 2/21 cases (in LOOCV), selected different sparsely overlapping feature profiles for the same classification task. From 267 unique features found in any of the 21 LOOCV sets, only 48 (18%) were common for five or more sets and none was common for all 21 sets. Previously, we found a similar variability in the diagnostic profiles when using a library of peptide IgM mimotopes [8].

Finding classifiers working well with the training set when using efficient feature selection algorithms is not so surprising. The space of subsets of more than 42 features out of 4524 is for all practical purposes infinite (10^102^), as is its capacity to produce false classifiers by overfitting to the training set. What is somewhat unexpected is that the uncovered diverse sets of features would together yield an accuracy of exact classification in LOOCV with such small training sets. It is likely that many diverse classifiers can be found for the same task. A possible explanation would be that the IgM repertoire readily provides different efficient classifiers, which would converge on a common public repertoire profile as the number of training sets increases. In other words, the data suggest that a number of partially overlapping “repertoire phenotypes” exist among individuals. Some of these “repertoire phenotypes” may be temporary states of the repertoire that oscillate in time [47]. At any point in time, combinations of increasing numbers of individual snapshot “repertoire phenotypes” would yield classifiers asymptotically approximating the global and dynamic public profile of the specificities relevant to the classification task. Such a scenario would also provide an opportunity for optimizing the “recombinations” of these profiles in order to obtain a classifier with an optimal generalization in the population. Overall, the efficiency of the classifiers based on actual tumor antigens seems higher than that based on mimotopes [8].

In the present study, we strived to address the problem of the interpretability of the IgM reactivity profiles. Namely, do actual peptides from known TAA and linear B cell epitopes provide more information when compared to arrays of random peptides [18] and synthetic mimotopes from phage display [8]? The readily accessible correlations with blood group antigen expression and the self/non-self nature of the epitopes confirmed this expectation. At the same time, it was not surprising that the IgM reactivities had little if any antigen specificity but rather related classes of cross-reactive structures found in different proteins. The reactivity graph provided insight into the structure of the IgM cross-reactivity repertoire, demonstrating the central role of blood group antigens (and maybe other widespread anti-carbohydrate) IgM reactivity and the non-self vs. self reactivities. Interestingly, the respective reactivity graph clusters helped extract sequence profiles that were also found in microbial protein antigens as demonstrated by the XSTREME algorithm. Thus, the reactivity graph demonstrated a possible link between various non-self reactivities including bacterial and viral proteins and blood group antigens, which could be a basis for immunological mimicry.

Interestingly, the most informative cross-reactivities in classifying patients with tumors were predominantly among weak self-reactivities separated from the blood group cross-reactivity dominated clusters. For their part, they also provided sequence profiles related to a wide variety of human or bacterial antigens including some expressed in the brain tissues and unrelated to the template tumor antigens used in testing array.

## 4. Materials and Methods

### 4.1. Patients’ Sera

The presented study includes surgically treated patients in the Clinic of Neurosurgery at St. Ivan Rilski University Hospital, Sofia, Bulgaria. All blood serum and brain tumor samples were obtained and analyzed after signed informed consent approved by the Ethics Committee at Medical University—Sofia. Only patients with proven glioblastoma or cerebral metastases of known origin were included. Patients who had undergone previous radio-, chemo- or immunotherapy, who suffered from an autoimmune disease or had an inflammatory disease in the past three months were excluded.

Sera were obtained from randomly selected patients with glioblastoma multiforme (GBM, n = 10), brain metastases of prostate or lung cancers (meta, n = 5), and non-tumor-bearing patients (contr, n = 6) with herniated disc surgery, trauma, etc. The sera were aliquoted and stored at −20°C. Before staining, the sera were thawed; incubated for 30 min at 37 °C for dissolution of IgM complexes; diluted 1:100 PBS, pH 7.4, 0.05% Tween 20 with 0.1% BSA, further incubated for 30 min at 37°C, and filtered through 0.22 μm filters before use.

### 4.2. Peptide Microarray

Standard tumor antigen microarray chips PEPperCHIP^®^ containing 4526 peptides produced by PEPperPRINT^TM^ (Heidelberg, Germany) were used. The peptides were synthesized in situ attached to the surface through their C-terminus and a common spacer GGGS and were included in the microarray layout in duplicate. The majority of the peptides represent a scan of the sequences of 24 tumor-associated antigens using a window of 15 amino acid residues shifted by two residues (Table 2). In addition, the peptide set contained peptides from 209 B cell epitopes tagged in the Immune Epitope Database (IEDB, [48]) “cancer” or “tumor” and coming from 28 viral antigens and plasminogen-binding protein pgbA from *Helicobacter pylori* (Table 3).

The chips were blocked for 60 min using PBS, pH 7.4, and 0.05% Tween 20 with 1% BSA on a rocker; washed 3 × 1 min with PBS, pH 7.4, and 0.05% Tween 20; and incubated with sera in dilutions equivalent to 0.01 mg/mL IgM (∼1:100 serum dilution) on a rocker overnight at 4 °C. After 3 × 1 min washing, the chips were incubated with secondary antibodies at room temperature (RT), washed, rinsed with distilled water, and dried by spinning in a vertical position in empty 50 mL test tubes at 100× *g* for 2 min.

### 4.3. Microarray Data Analysis

The microarray images were acquired using a GenePix 4000 Microarray Scanner (Molecular Devices, USA). The densitometry was done using GenePix R Pro v6.0 software. All further analysis was performed using publicly available packages of the R statistical environment for Windows (v3.4.1) (Bioconductor; Biostrings, limma, pepStat, sva, e1071, Rtsne, clvalid, etc.) as well as in-house developed R scripts (https://github.com/ansts/TAAIgM, last accessed 31 December 2022). The details of the analysis procedures are described elsewhere [8].

Graph studies were based on the functions in the igraph package, while the visualization was done in Gephi software after exporting the graph with all vertex attributes in a graphml format. Different properties of peptide reactivities, cliques, and neighborhoods were compared using linear mixed effects model or nested ANOVA.

The feature selection algorithm consisted of a filter and a wrapper stage. In the filter stage, the 3- and 4-cliques of the graph were each tested as a subspace of the 4512-dimensional reactivity space for their capacity to map the three diagnostic groups to well separable clusters. Those with clustering criterion (see below) better than the 95th percentile of the distribution of the criterion calculated from mappings on random samples of reactivities of the same dimensionality (the same number of 3 or 4 peptides) were filtered as candidates for the classifier.

The selected features were further subjected to feature selection by recursive elimination. The criterion for selection was based on dichotomous supervised clustering of the patients in the different diagnostic groups (GBM vs. Control plus Metastatic or Control vs. tumor bearing). The goal was optimal clustering of the predetermined diagnostic groups estimated using a composed criterion based on 3 internal clustering indices: Baker–Hubert gamma [49], Dunn [50,51], and connectivity [51] (for details see the supplemental methods of [8]). This algorithm allowed an incremental improvement of the quality of the clustering by recursive feature elimination of features one at a time. It yielded a feature subset with a greatly improved capacity to classify the diagnostic groups. Three selection steps were performed, each dichotomously differentiating one of the diagnostic groups from the rest, and the resulting feature sets were pooled, selecting only features that appeared in at least 2 of the steps.

The significance of the approach and the selected feature sets were validated in a “leave-one-out” cross-validation (LOOVC) scheme encompassing the whole algorithm staring with the generation of the graph. Thus, the algorithm of a filter and a wrapper stage of the feature selection described above was applied successively to all sets of 20 patients excluding in turn each of them, and the capacity of the resulting feature set to predict correctly the excluded case was tested using a support vector machine model of the data projected to 2 dimensions using multidimensional scaling. The criteria for the efficiency of the classifier were F1 and Matthew’s clustering criterion calculated on the basis of the confusion matrix of prediction versus ground truth. Once the algorithm was validated, the ultimate model was constructed from features that recurred in the feature sets from the LOOCV. The threshold for the number of recurrences was determined empirically by scanning the different thresholds with the same clustering criterion.

## 5. Conclusions

The proposed algorithm for the extraction of IgM repertoire-based diagnostic classifiers has the potential to provide a fast, non-invasive, and inexpensive tool for the analysis of malignant tumors. By using natural peptides from known tumor-associated antigens, it is possible to find interpretable reactivities pointing to known or unknown prognostic markers, which would give new opportunities in the diagnosis and treatment with possibly wider application in tumor immunology.

## Figures and Tables

**Table 3:** Viral (bacterial) antigen epitopes, of which the linear fragments are included in the study.

	Protein		N
HHV4 (EBV)	BZLF1 Human herpesvirus 4	EBV-BZLF1	79
	Capsid protein VP26 Human herpesvirus 4 (strain B95-8)	EBV_VP26	11
	Early antigen protein R Human herpesvirus 4 (strain B95-8)	EBV_EA	3
	Epstein–Barr nuclear antigen 1 Human herpesvirus 4	EBV_EBNA1	45
	Latent membrane protein 2 Human herpesvirus 4 (strain B95-8)	EBV_LMP2	3
	ribonucleoside-diphosphate reductase large chain Human herpesvirus 4	EBV_RIR1	8
HPV ( types 6, 16, 18, and 33)	E2 protein Human papillomavirus type 6	HPV6_E2	2
	major capsid protein Human papillomavirus type 6	HPV6_L1	6
	L2 Human papillomavirus type 6	HPV6_L2	4
	Replication protein E1 Human papillomavirus type 16	HPV16_E1	6
	E2 protein Human papillomavirus type 16	HPV16_E2	9
	Probable protein E4 Human papillomavirus type 16	HPV16_E4	22
	E6 protein Human papillomavirus type 16	HPV16_E6	10
	E7 protein Human papillomavirus type 16	HPV16_E7	38
	L1 protein Human papillomavirus type 16	HPV16_L1	53
	L2 Human papillomavirus type 16	HPV16_L2	12
	E2 Human papillomavirus type 18	HPV18_E2	2
	E7 protein Human papillomavirus type 18	HPV18_E7	6
	L1 Human papillomavirus type 33	HPV33_L1	4
	Envelope glycoprotein gp62 precursor Human T-lymphotropic virus 1	HTLV1_env	125
HTLV1 and 2	Pr gag-pro-pol Human T-lymphotropic virus 1	HTLV1_gag	30
	rex protein Human T-lymphotropic virus 1	HTLV1_rex	10
	tax protein Human T-lymphotropic virus 1	HTLV1_tax	12
	envelope glycoprotein Human T-lymphotropic virus 2	HTLV2_env	11
HBV	Hbx protein Hepatitis B virus	HBV_X	13
HHV8	K8.1 Human herpesvirus 8	HHV8_K8_1	7
	LANA Human herpesvirus 8	HHV8_LANA	26
	small viral capsid antigen Human herpesvirus 8	HHV8_ORF65	21
H. pylori	Plasminogen-binding protein pgbA *Helicobacter pylori*	Hp_pgbA	1

## Data Availability

All data and scripts are available at https://github.com/ansts/TAAIgM, (accessed on 31 December 2022).

## Supplementary Materials

The following supporting information can be downloaded at: https://www.mdpi.com/article/10.3390/ijms24032597/s1.

## Abbreviations

GBM—glioblastoma multiforme, CSF—cerebrospinal fluid, TAA—tumor-associated antigens, HTLV-1—human T-lymphotropic virus, HPV-16—human papillomavirus 16, PBS—phosphate-buffered saline, BSA—bovine serum albumin, MCC—Mathew’s correlation coefficient, MMP11—matrix metalloproteinase, PSA—prostate-specific antigen, KLK—kallikrein, ErbB2—erythroblastic leukemia viral oncogene homolog B2.
