# Supplementary material for: Reactivity Graph Yields Interpretable IgM Repertoire Signatures as Potential Tumor Biomarkers
Source: Int J Mol Sci. 2023 Jan 30;24(3):2597. doi: 10.3390/ijms24032597 (PMC9917253; doi:10.3390/ijms24032597)

SVM_SupplFig_1.pdf

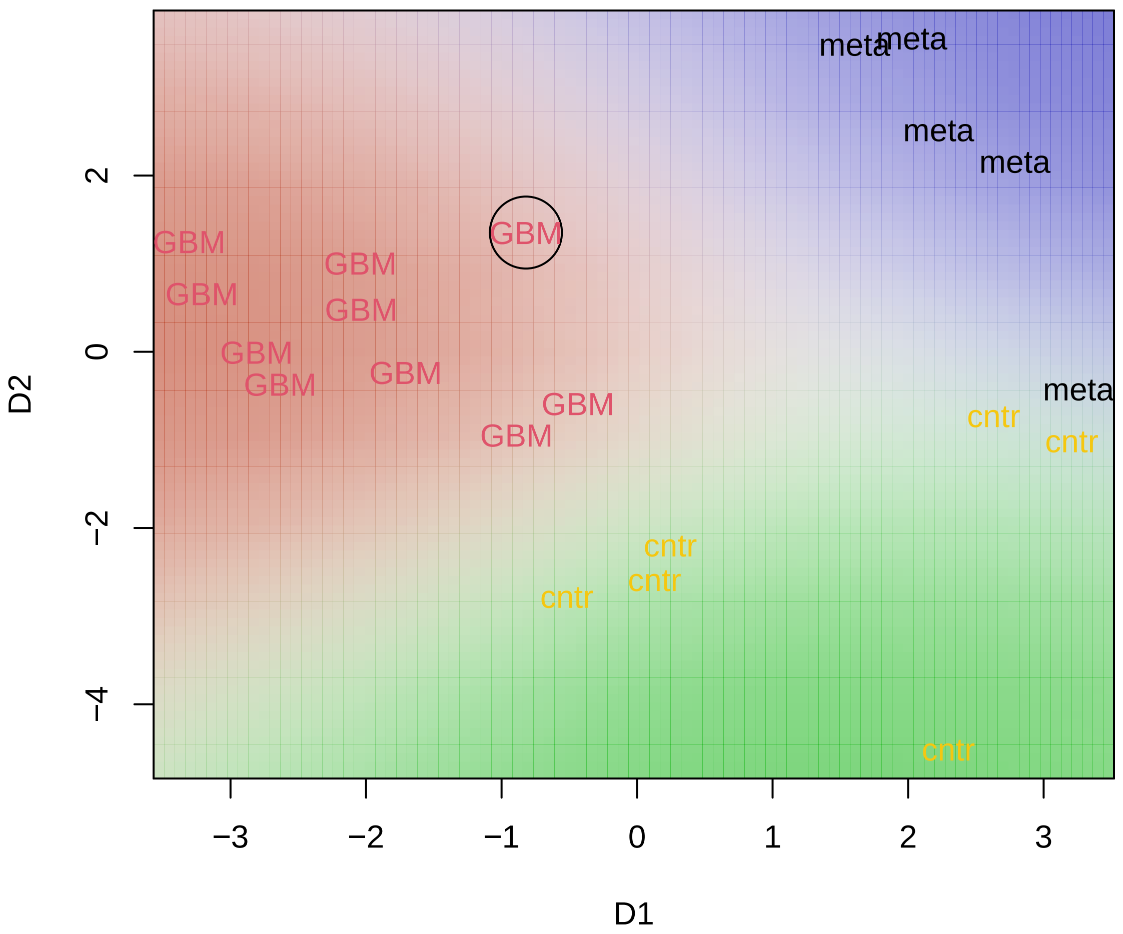

SVM_SupplFig_2.pdf

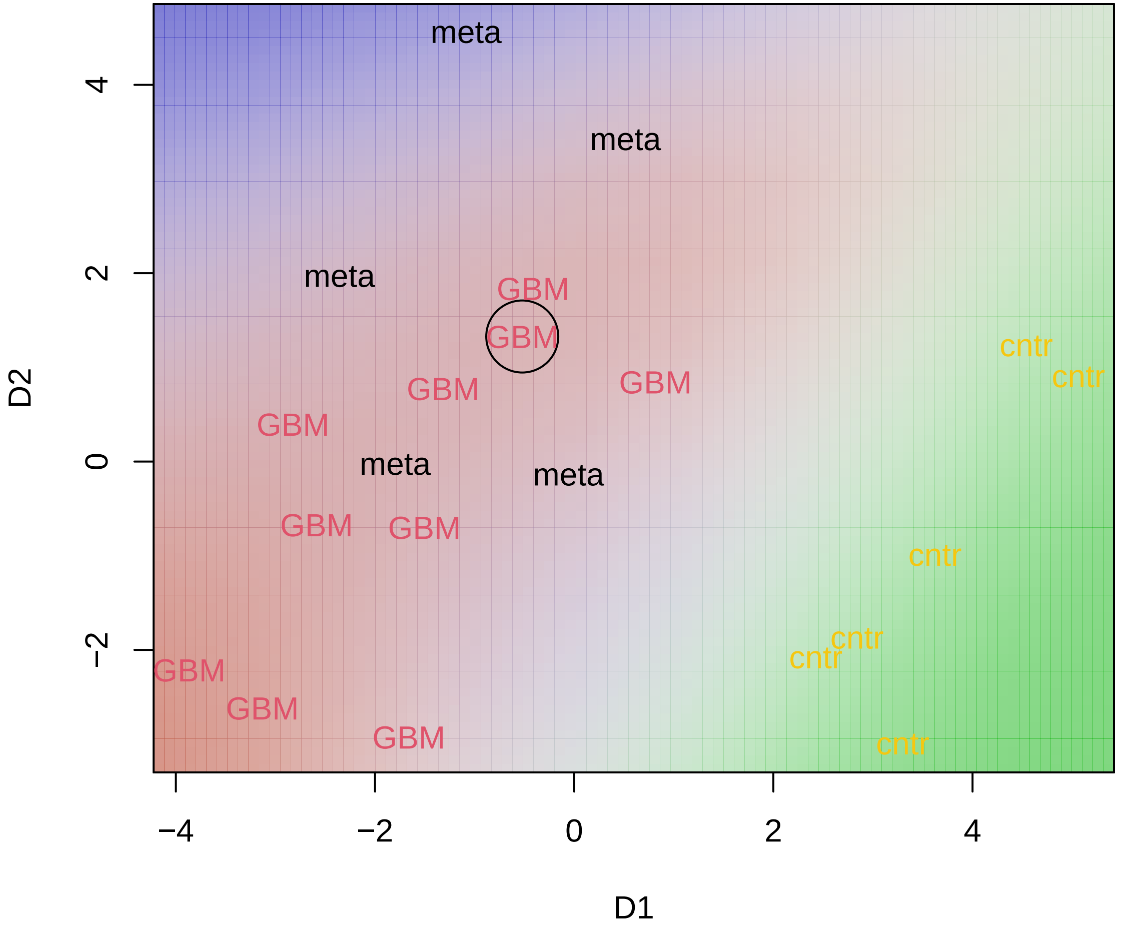

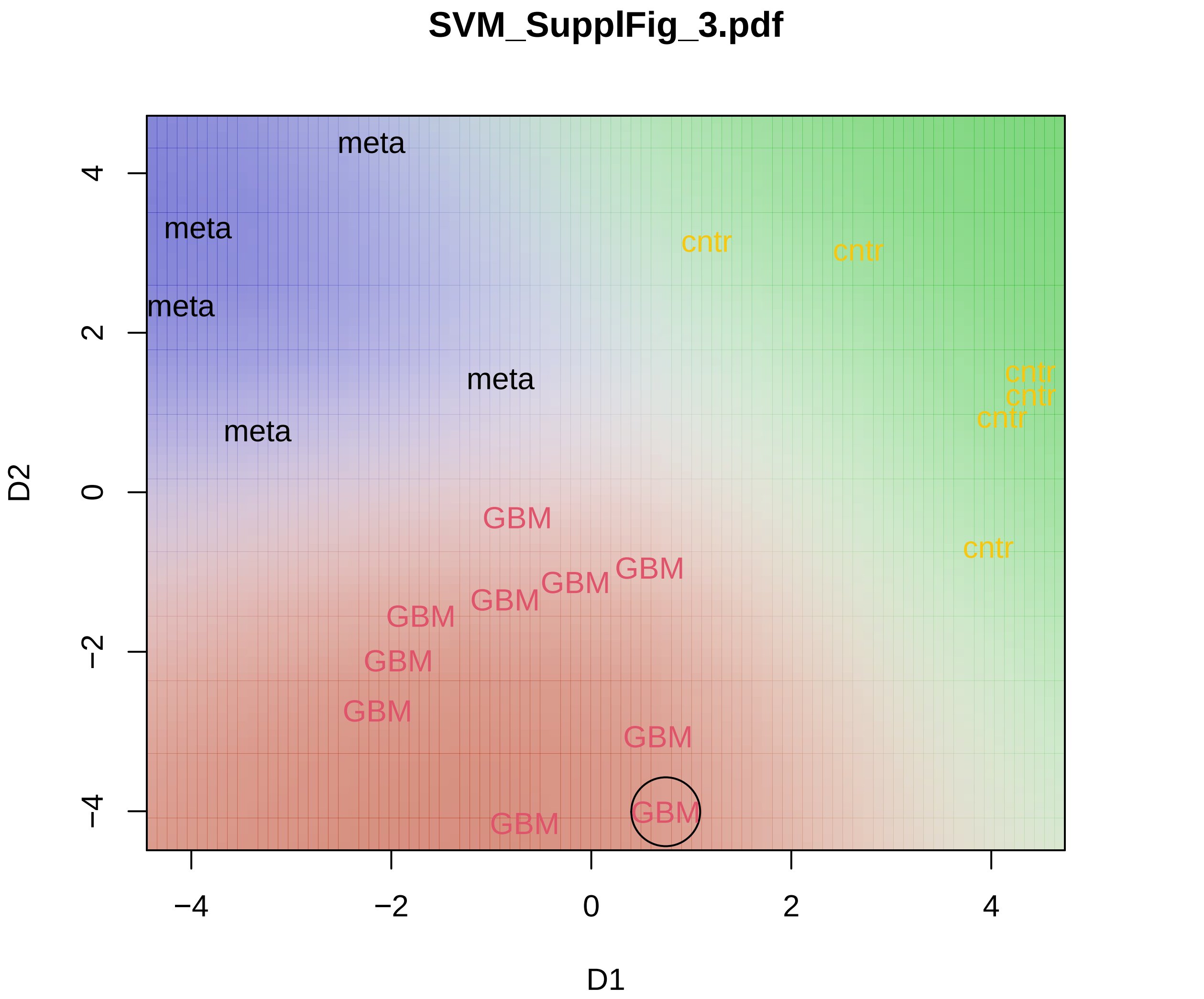
SVM_SupplFig_3.pdf
meta
meta
meta
meta
meta
cntr
cntr
cntr
cntr
cntr
cntr
GBM
GBM
GBM
GBM
GBM
GBM
GBM
GBM
GBM
GBM
D1
D2
−4
−2
0
2
4

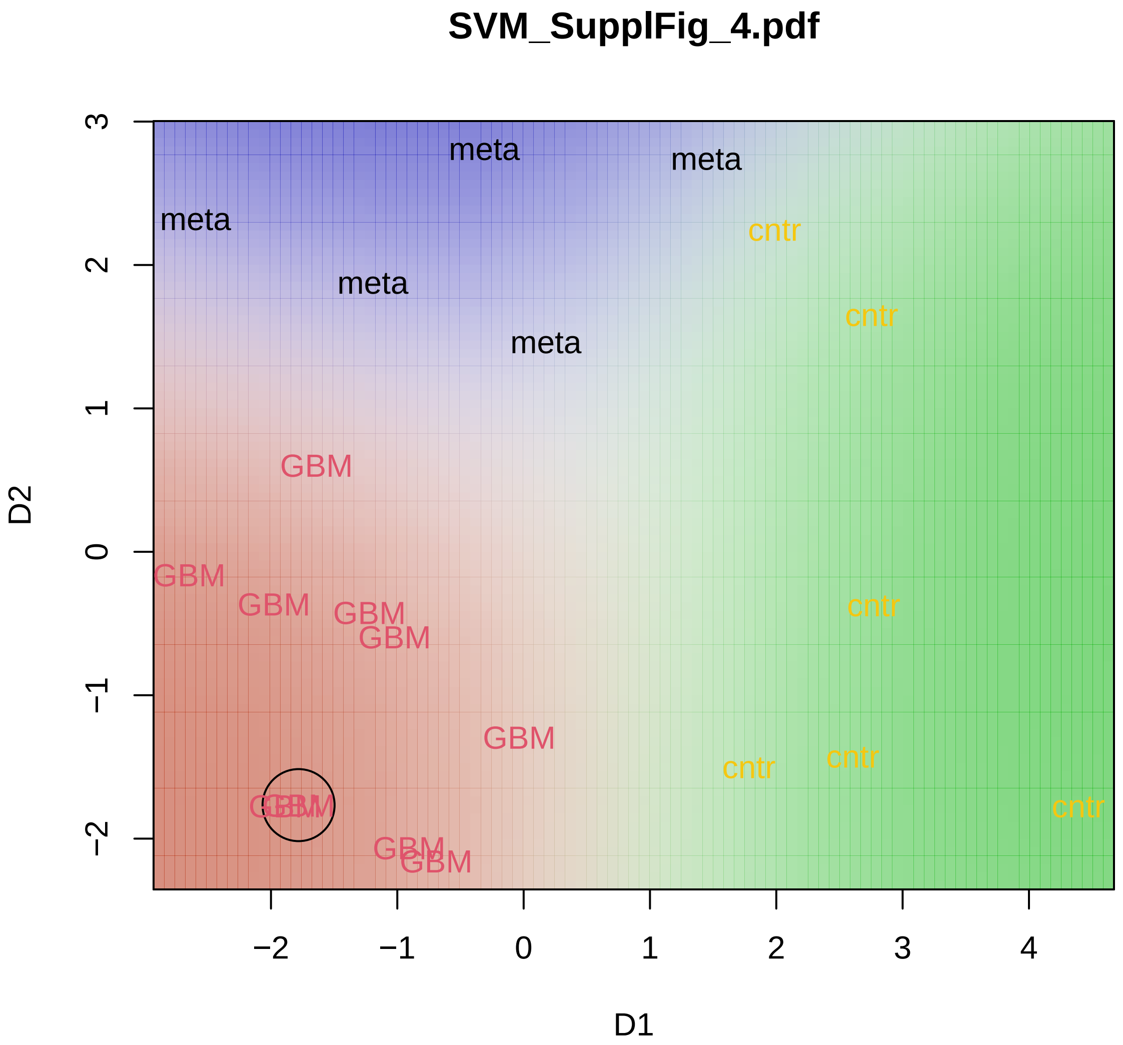
SVM_SupplFig_4.pdf
meta
meta
meta
cntr
meta
cntr
meta
GBM
GBM
GBM
GBM
GBM
cntr
GBM
cntr
cntr
cntr
GBM
GBM
D1
D2
−2
−1
0
1
2
3
4

SVM_SupplFig_5.pdf

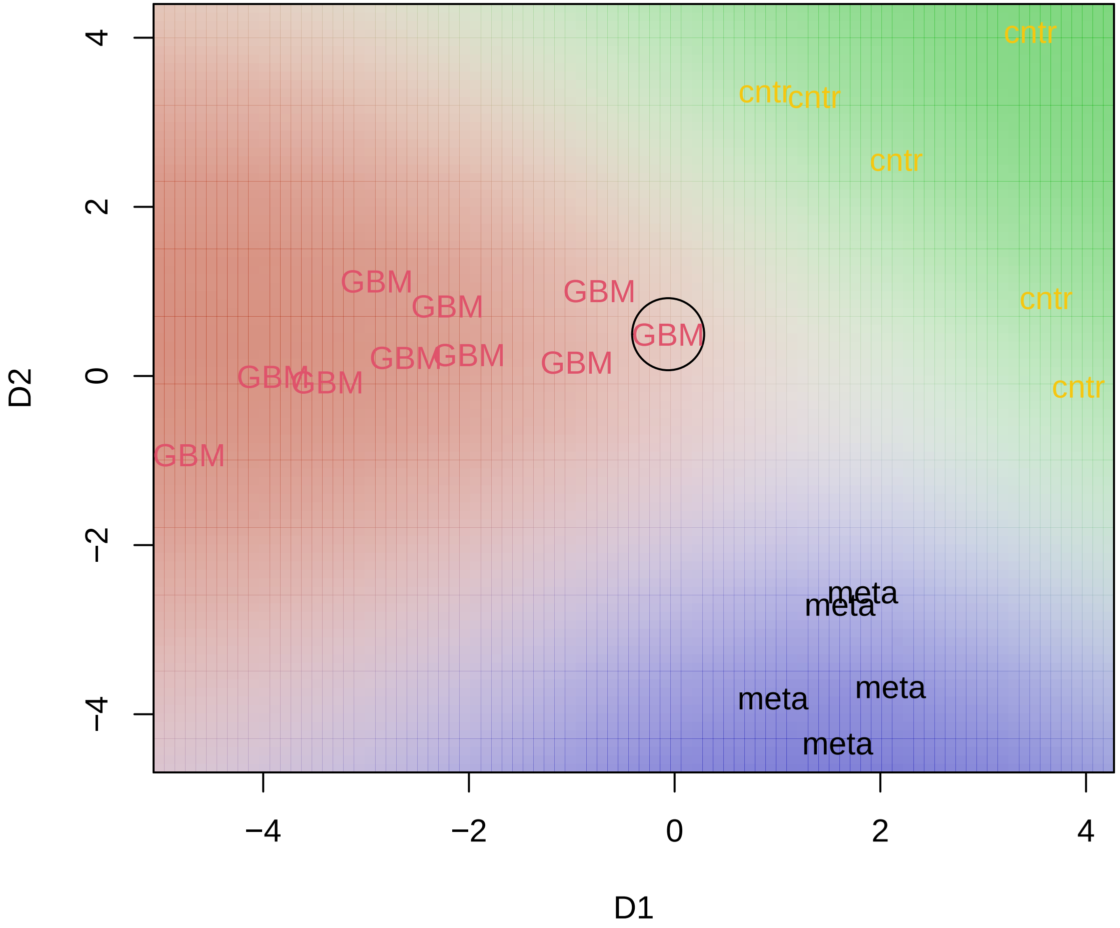

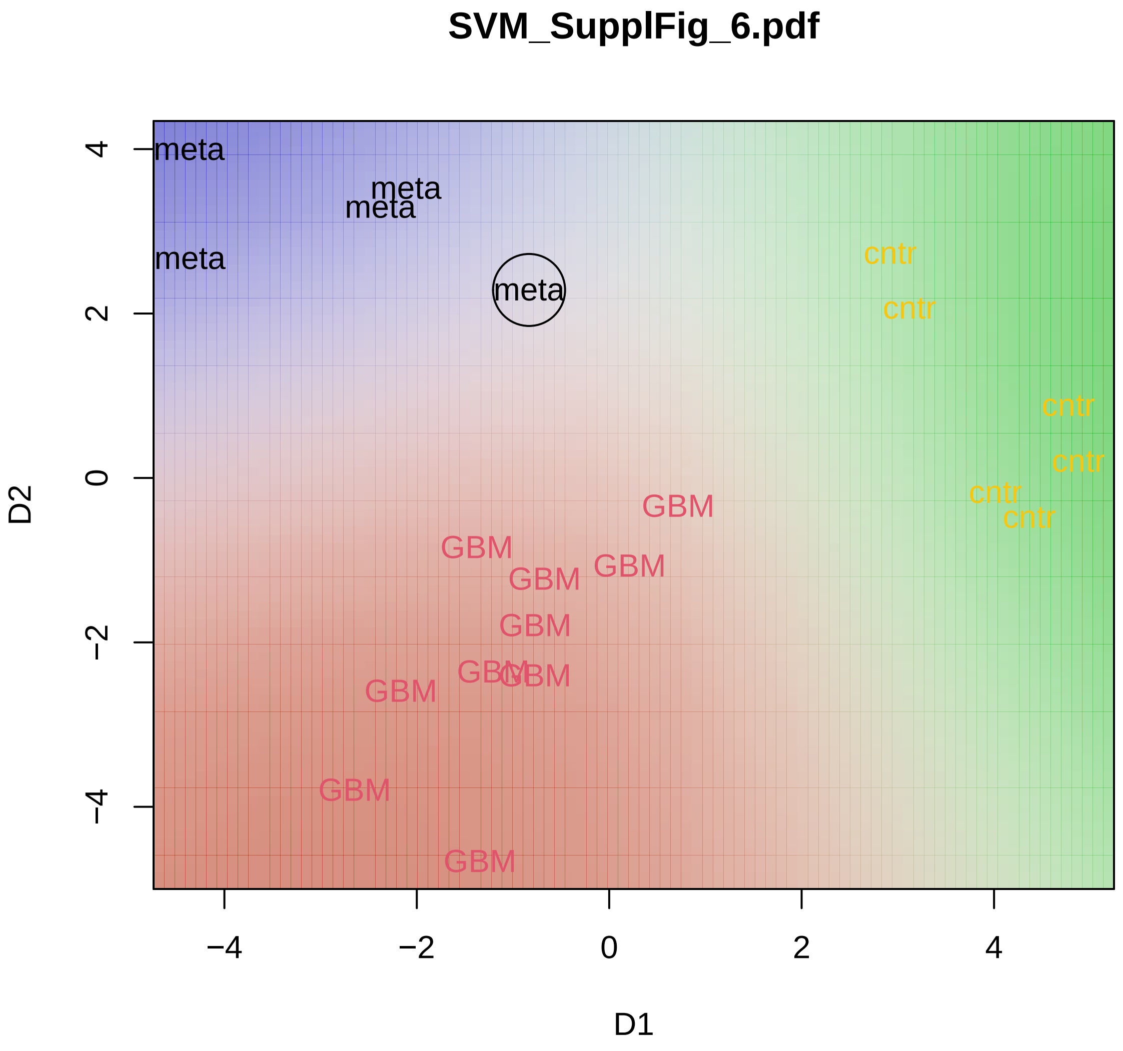
SVM_SupplFig_6.pdf
D1
D2
−4
−2
0
2
4
meta
cntr
GBM

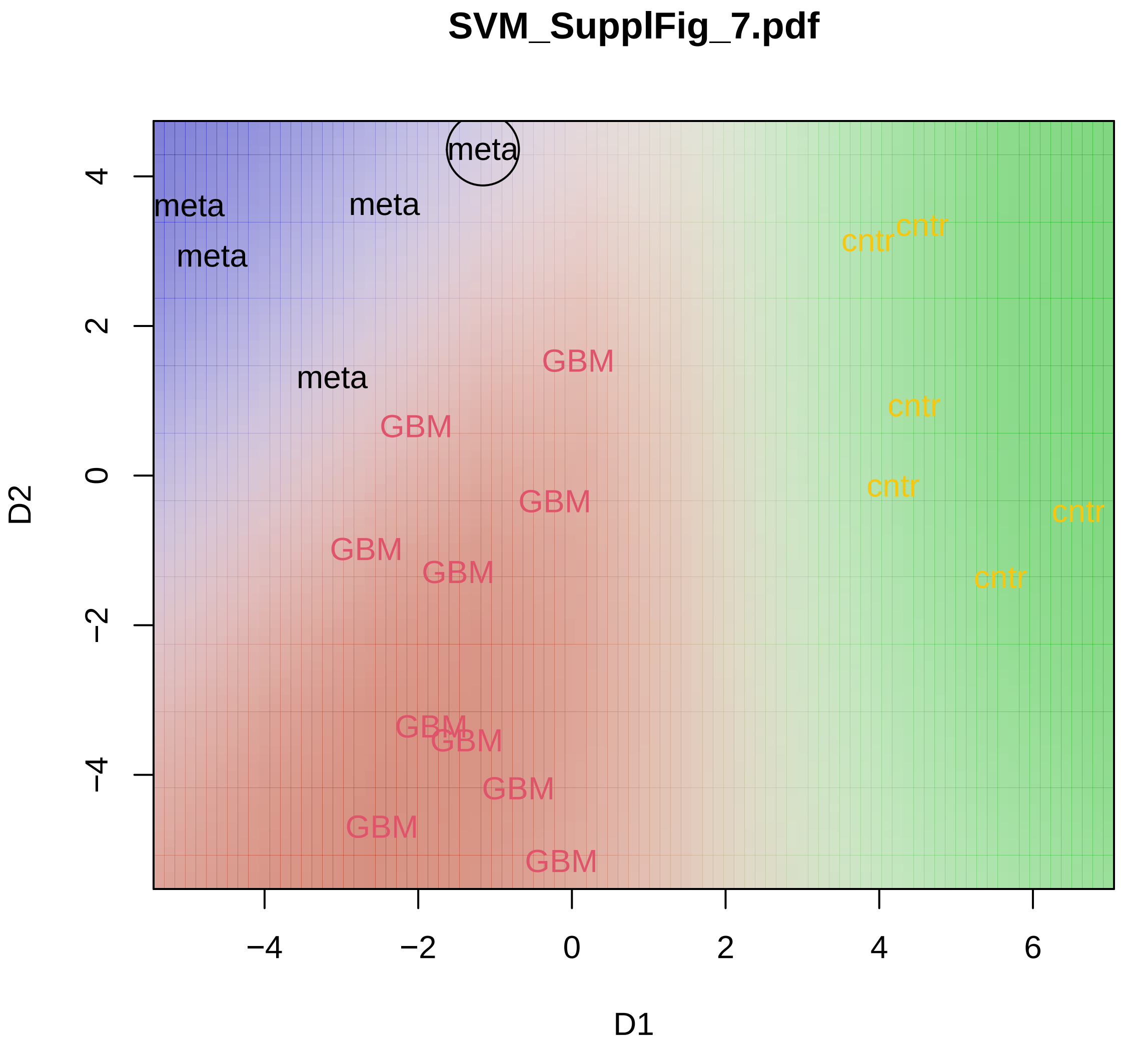
SVM_SupplFig_7.pdf
D1
D2
−4
−2
0
2
4
6
meta
meta
meta
meta
meta
GBM
GBM
GBM
GBM
GBM
GBM
GBM
GBM
GBM
GBM
cntr
cntr
cntr
cntr
cntr
cntr

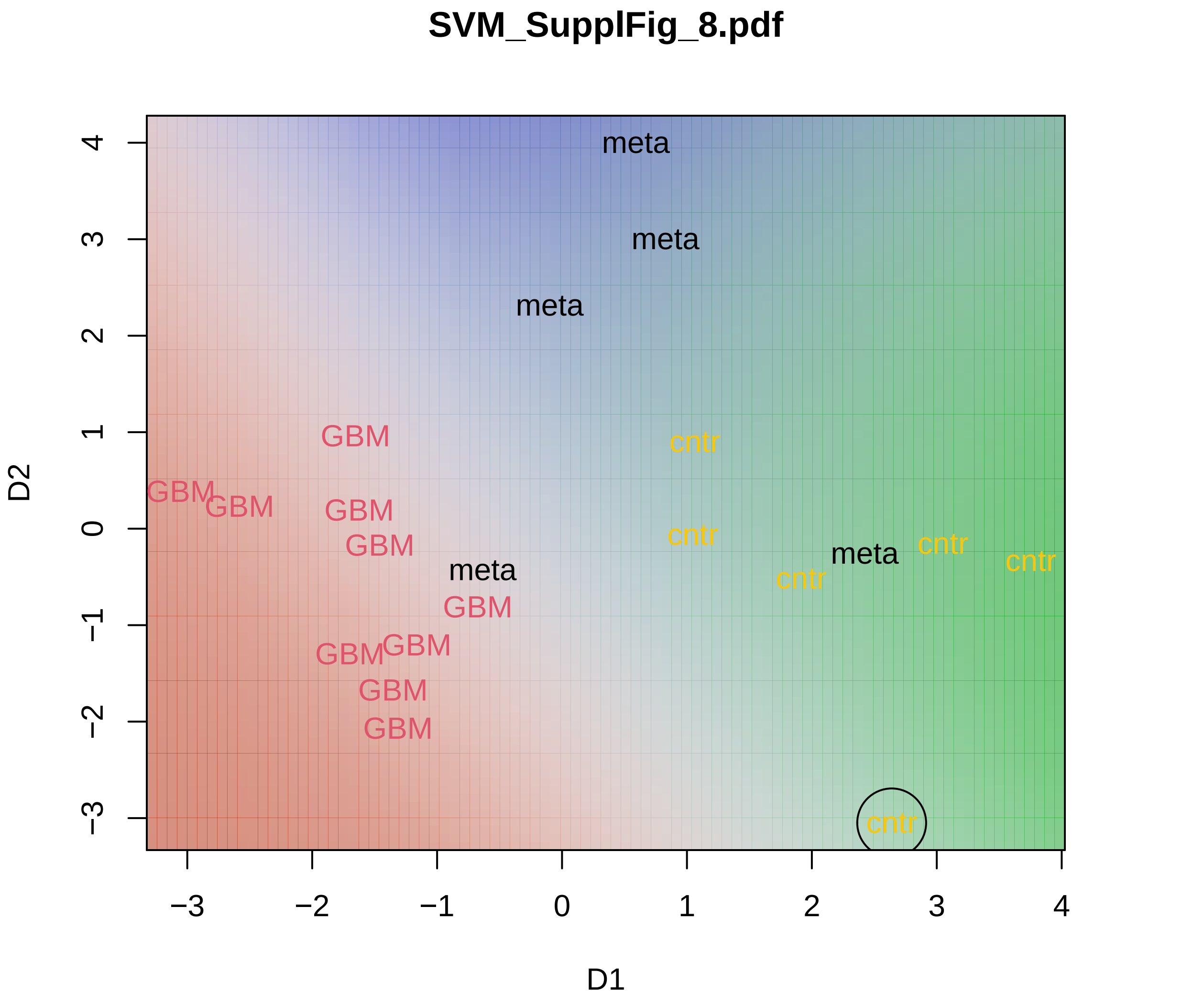
SVM_SupplFig_8.pdf
D1
D2
−3
−2
−1
0
1
2
3
4
meta
meta
meta
meta
meta
GBM
GBM
GBM
GBM
GBM
GBM
GBM
GBM
GBM
GBM
GBM
cntr
cntr
cntr
cntr
cntr
cntr

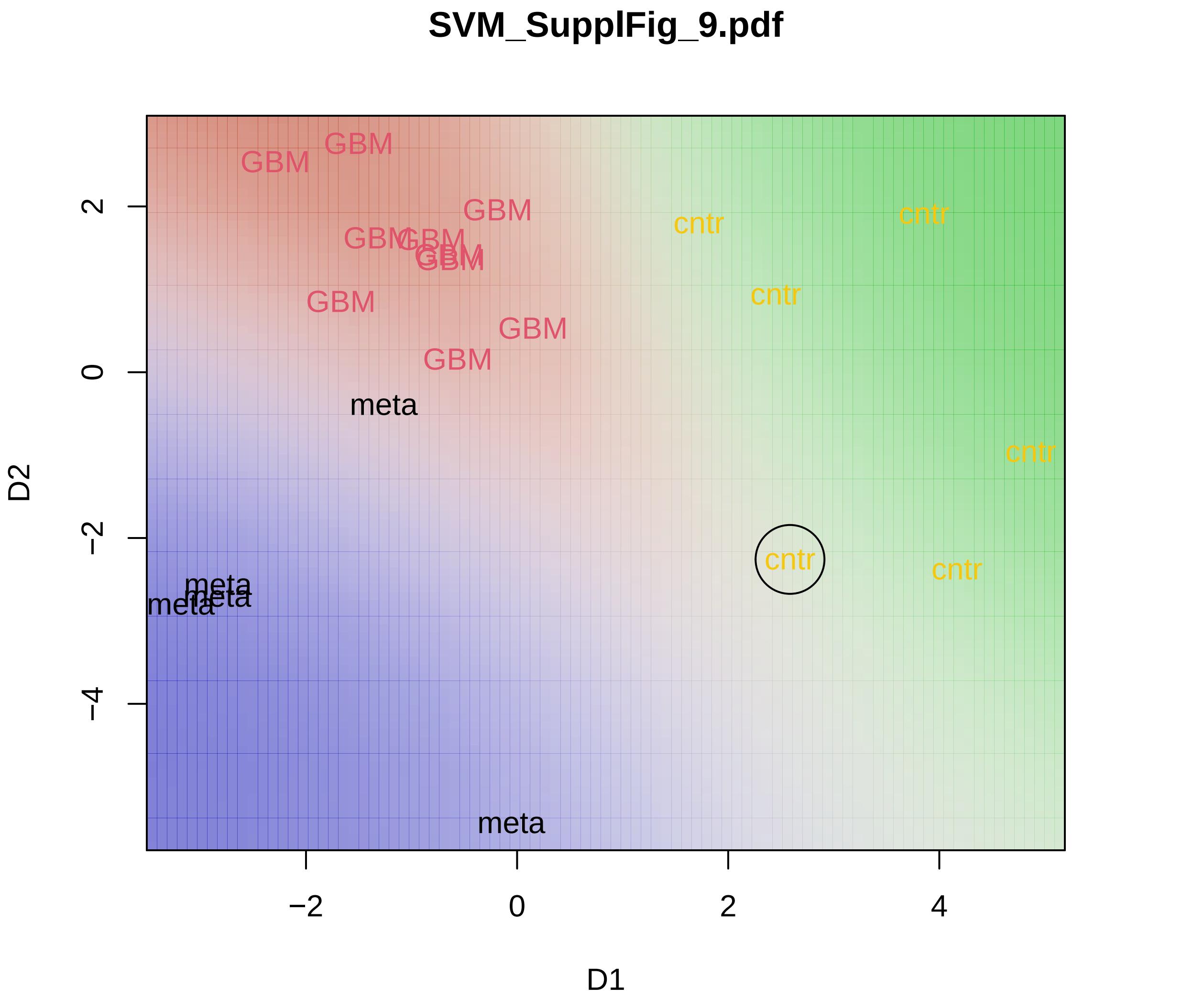
SVM_SupplFig_9.pdf
GBM
GBM
GBM
GBM
GBM
GBM
GBM
GBM
GBM
GBM
cntr
cntr
cntr
cntr
cntr
cntr
meta
meta
meta
meta
meta
D2
2
0
−2
−4
D1
−2
0
2
4

SVM_SupplFig_10.pdf

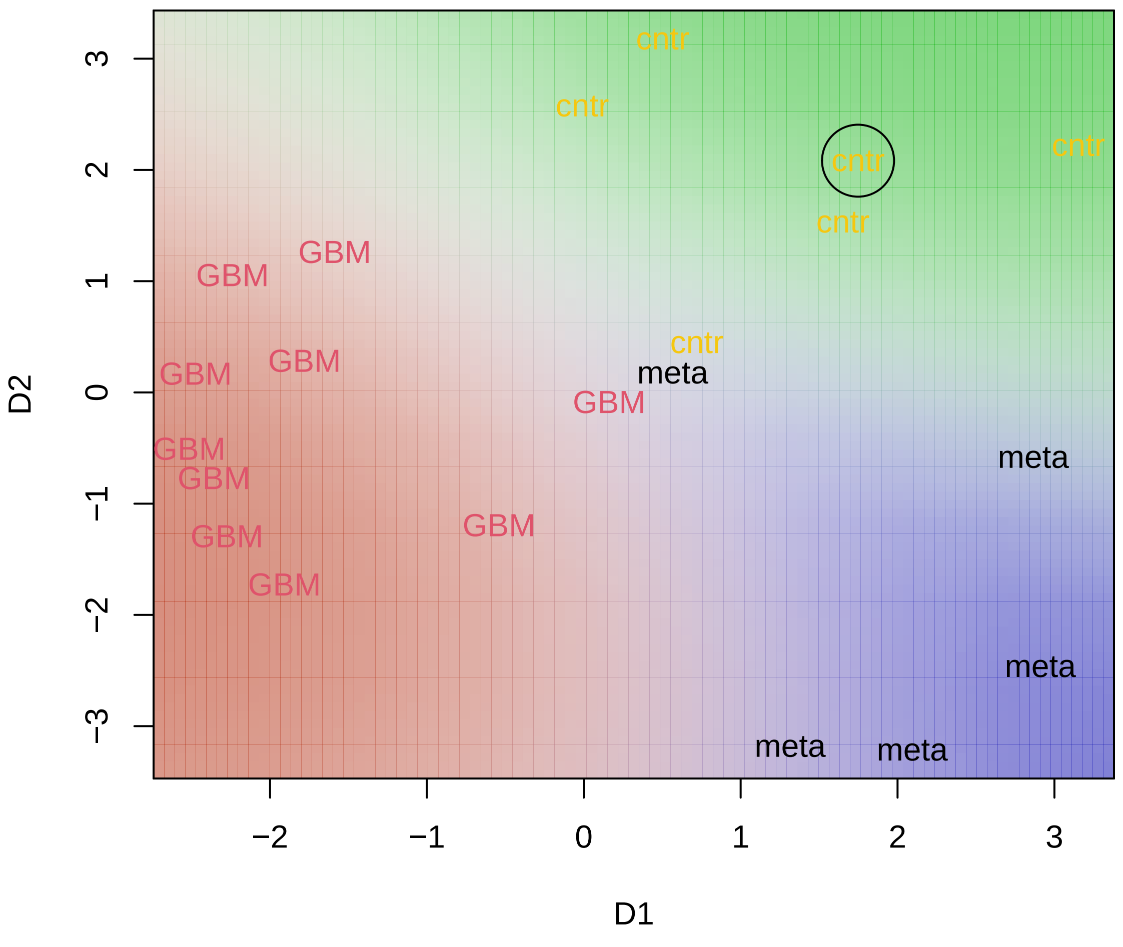

SVM_SupplFig_11.pdf

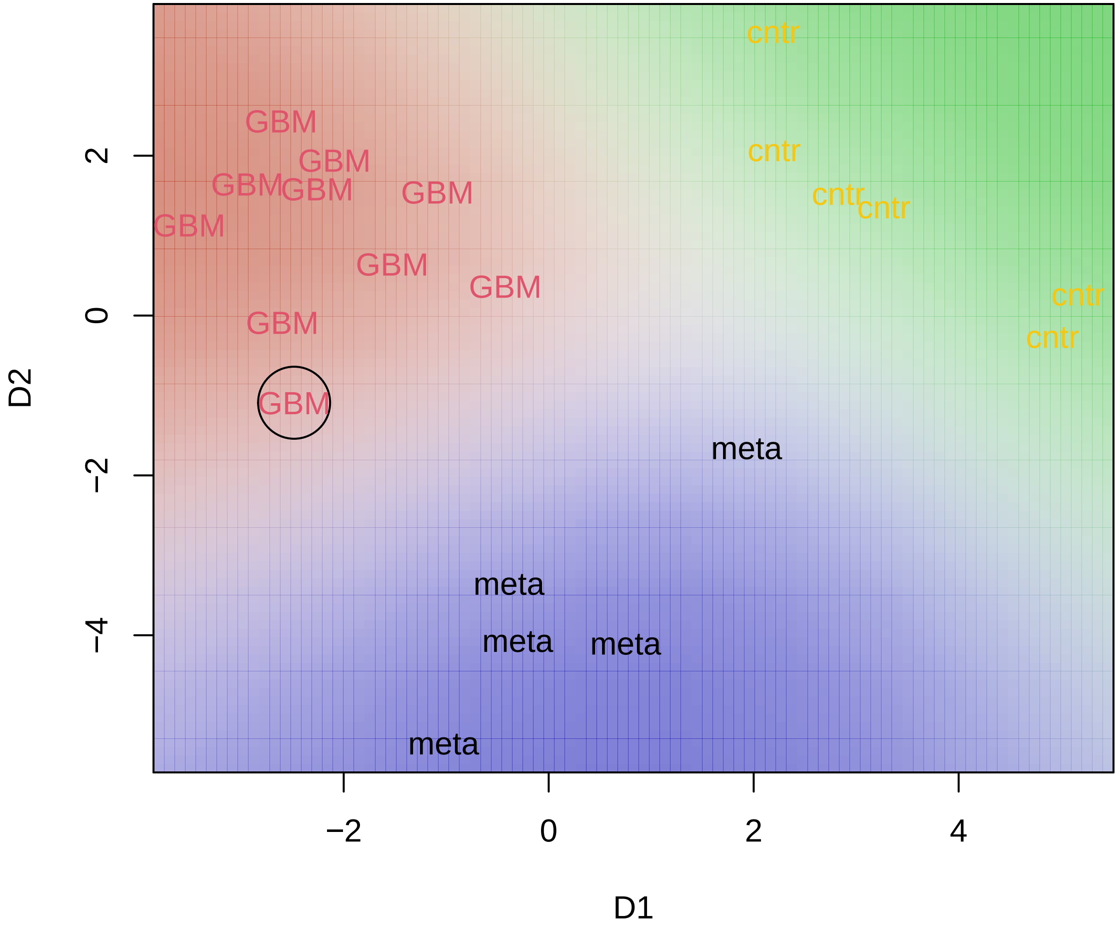

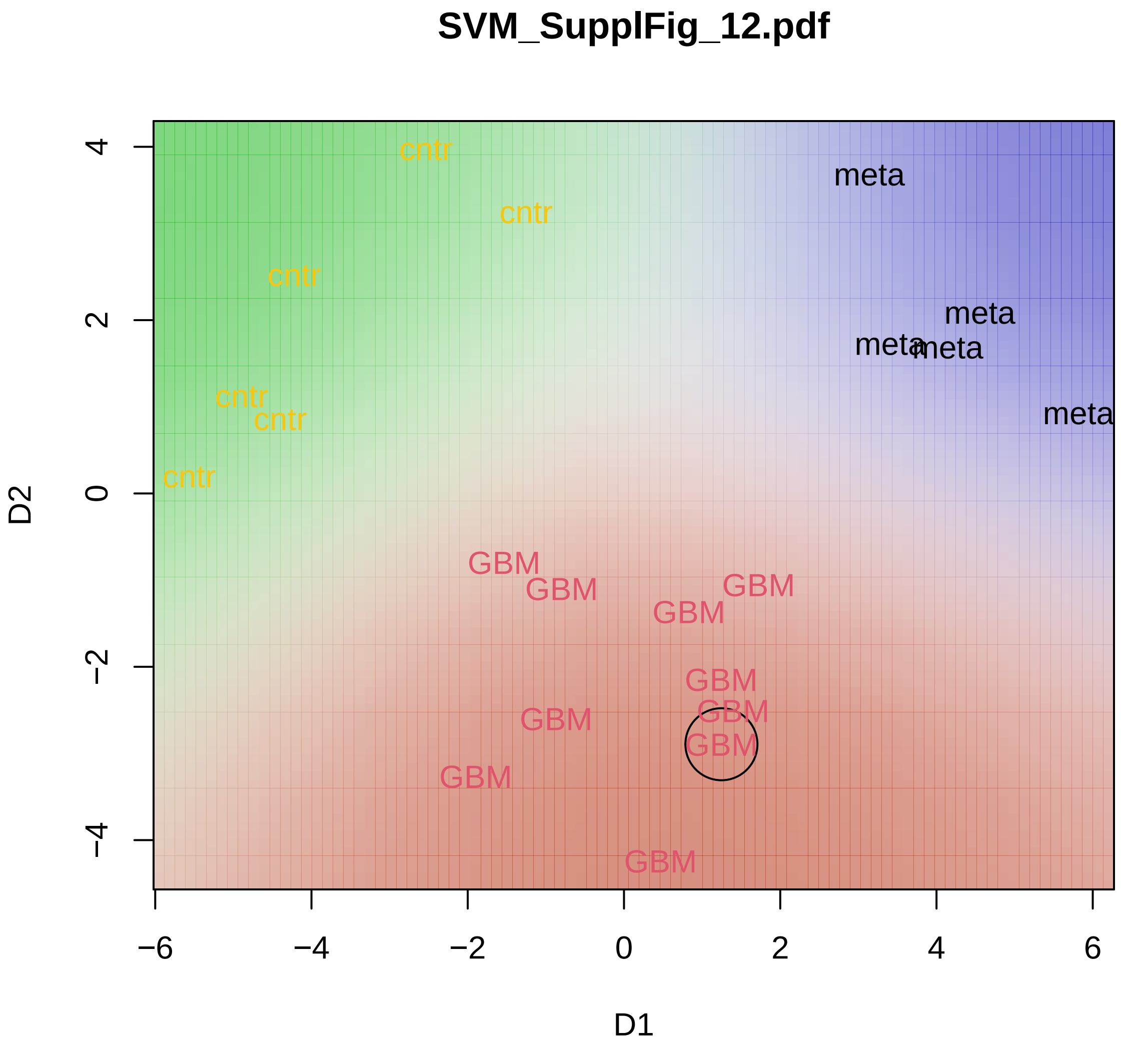
SVM_SupplFig_12.pdf
cntr
cntr
cntr
cntr
cntr
cntr
meta
meta
meta
meta
meta
GBM
GBM
GBM
GBM
GBM
GBM
GBM
GBM
GBM
GBM
D1
D2
−6
−4
−2
0
2
4
6
−4
−2
0
2
4

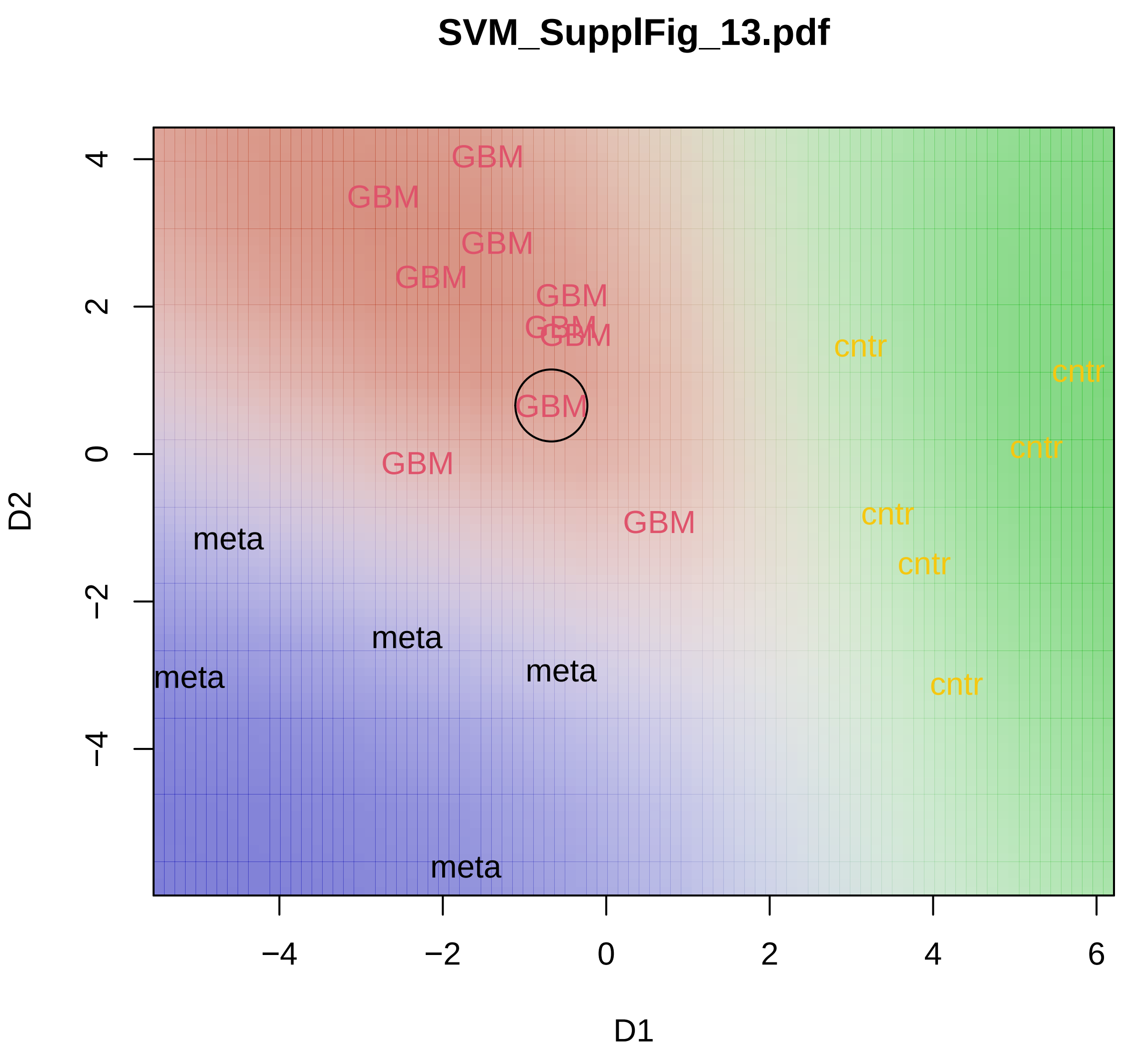

SVM_SupplFig_13.pdf
GBM
GBM
GBM
GBM
GBM
GBM
GBM
GBM
GBM
GBM
cntr
cntr
cntr
cntr
cntr
cntr
meta
meta
meta
meta
meta
4
2
0
−2
−4
−4
−2
0
2
4
6
D1
D2

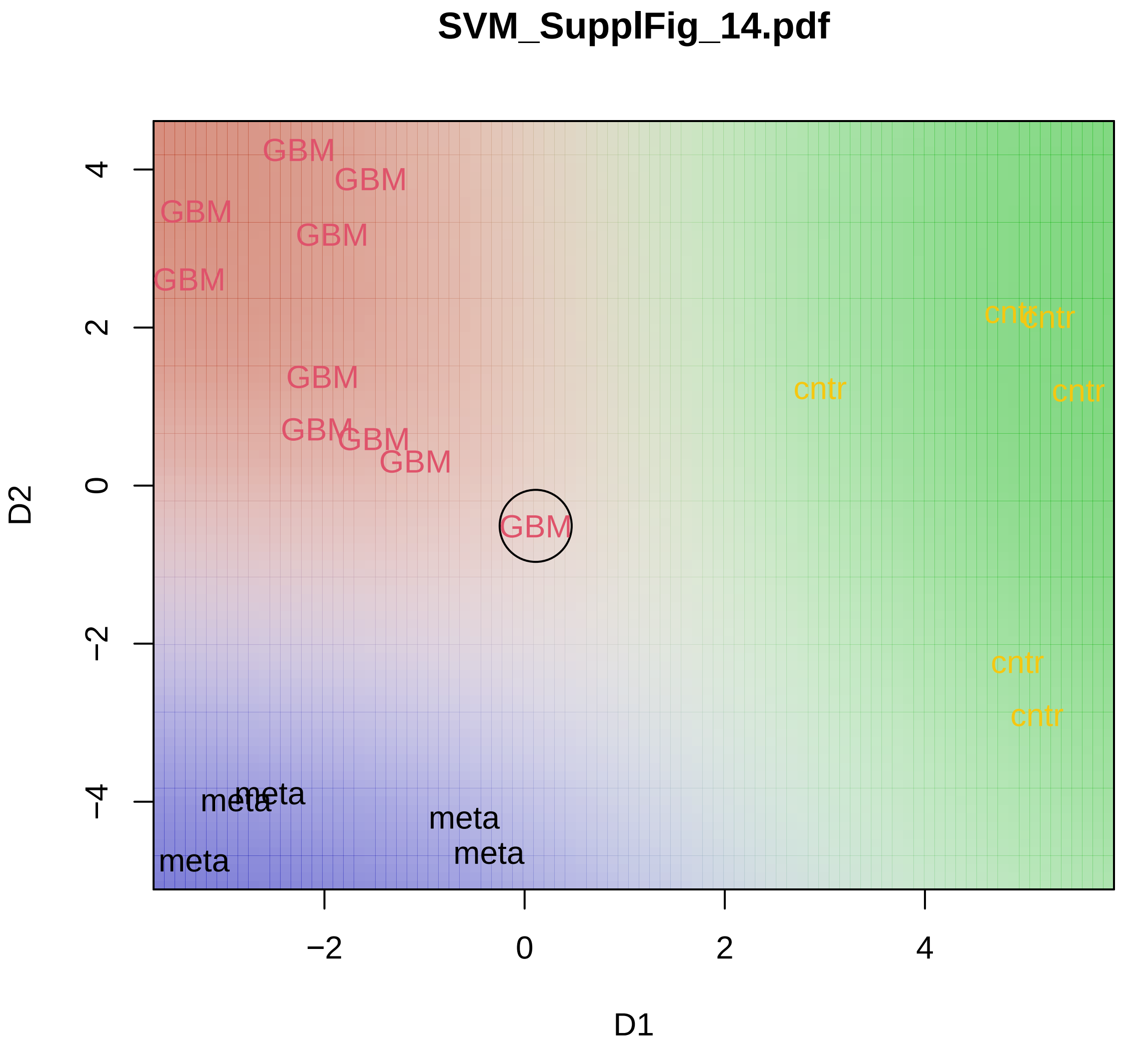
SVM_SupplFig_14.pdf
D1
D2
GBM
cntr
meta
−2
0
2
4
−4

SVM_SupplFig_15.pdf

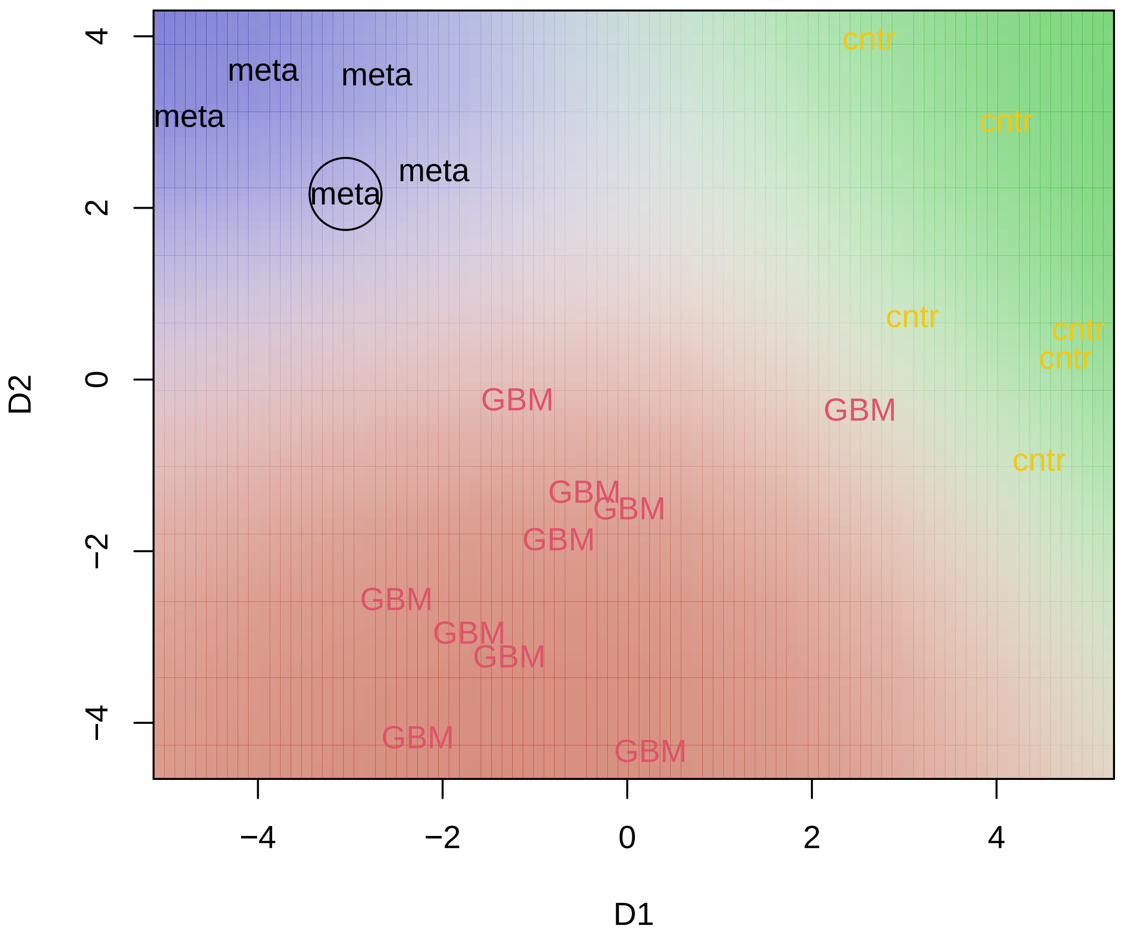

SVM_SupplFig_16.pdf

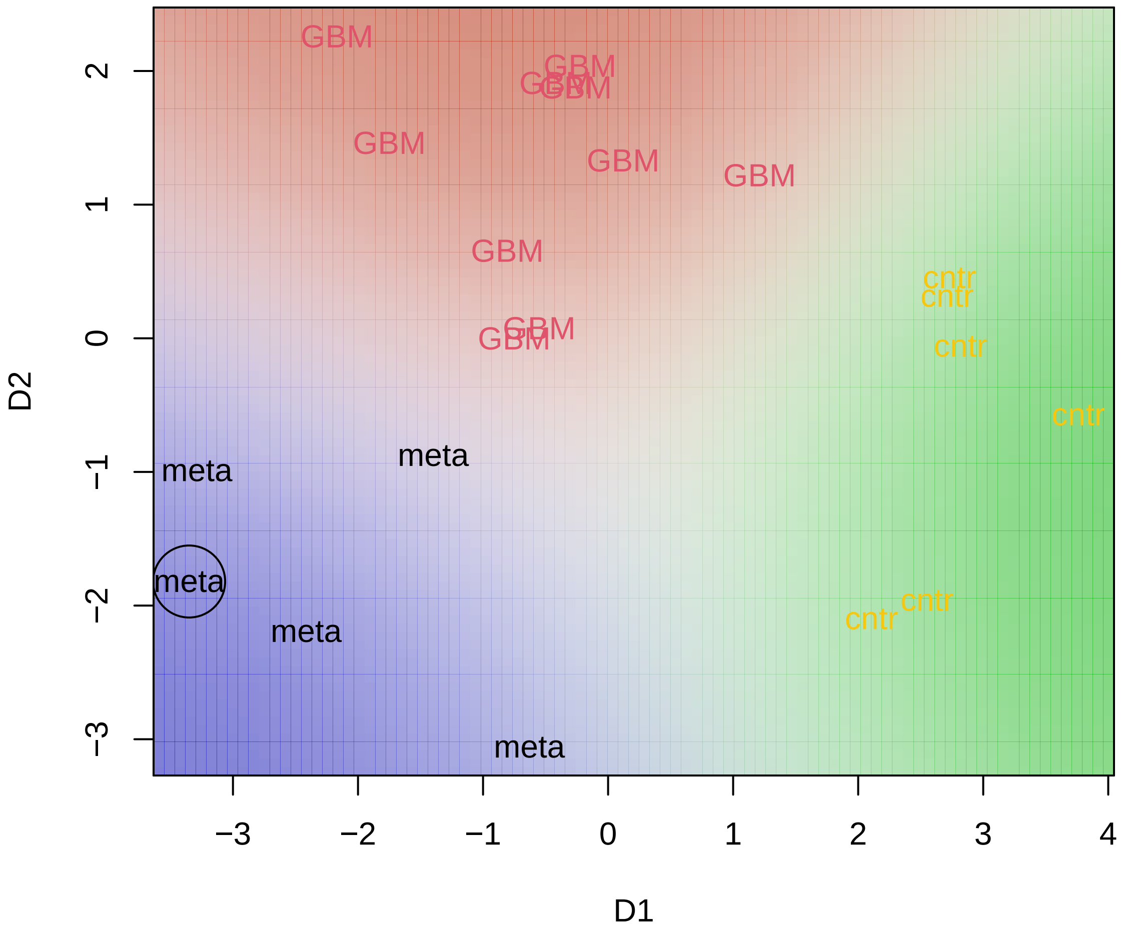

SVM_SupplFig_17.pdf

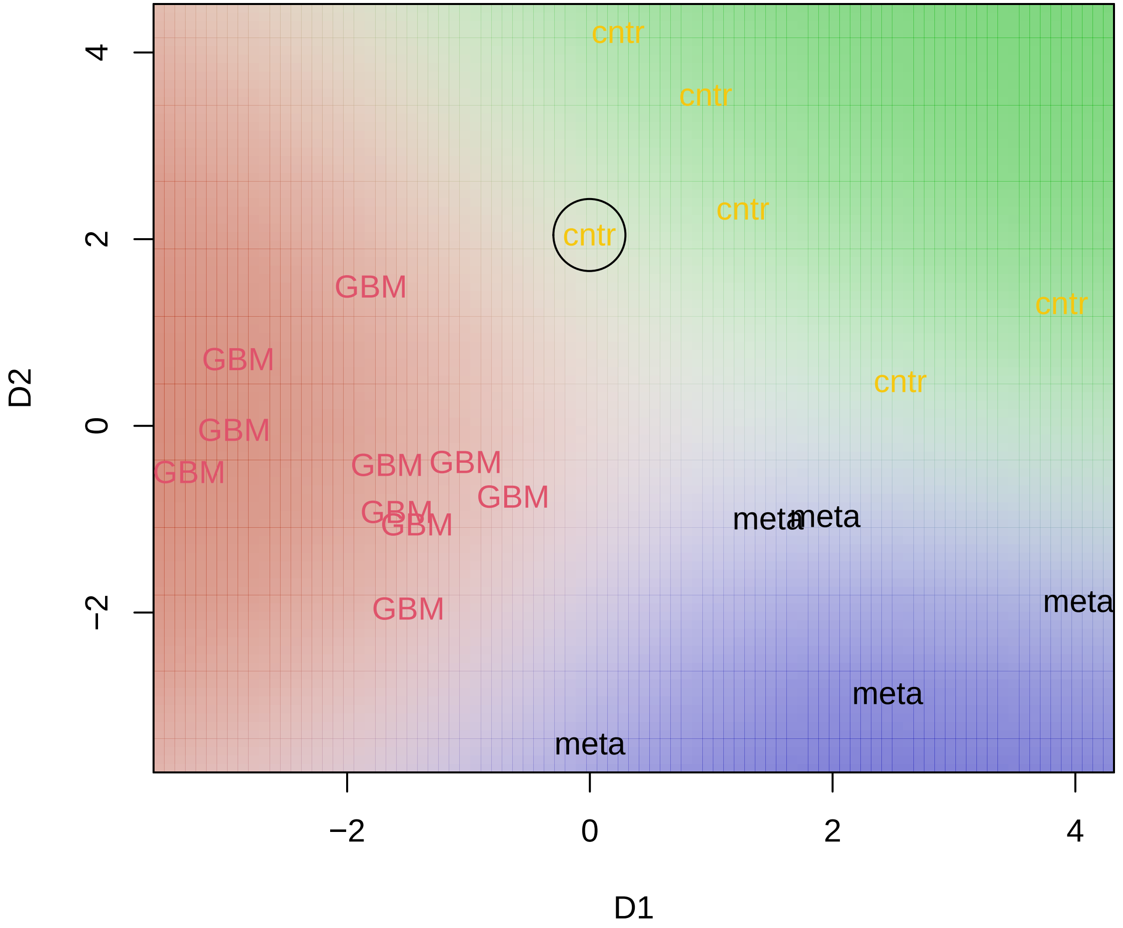

SVM_SupplFig_18.pdf

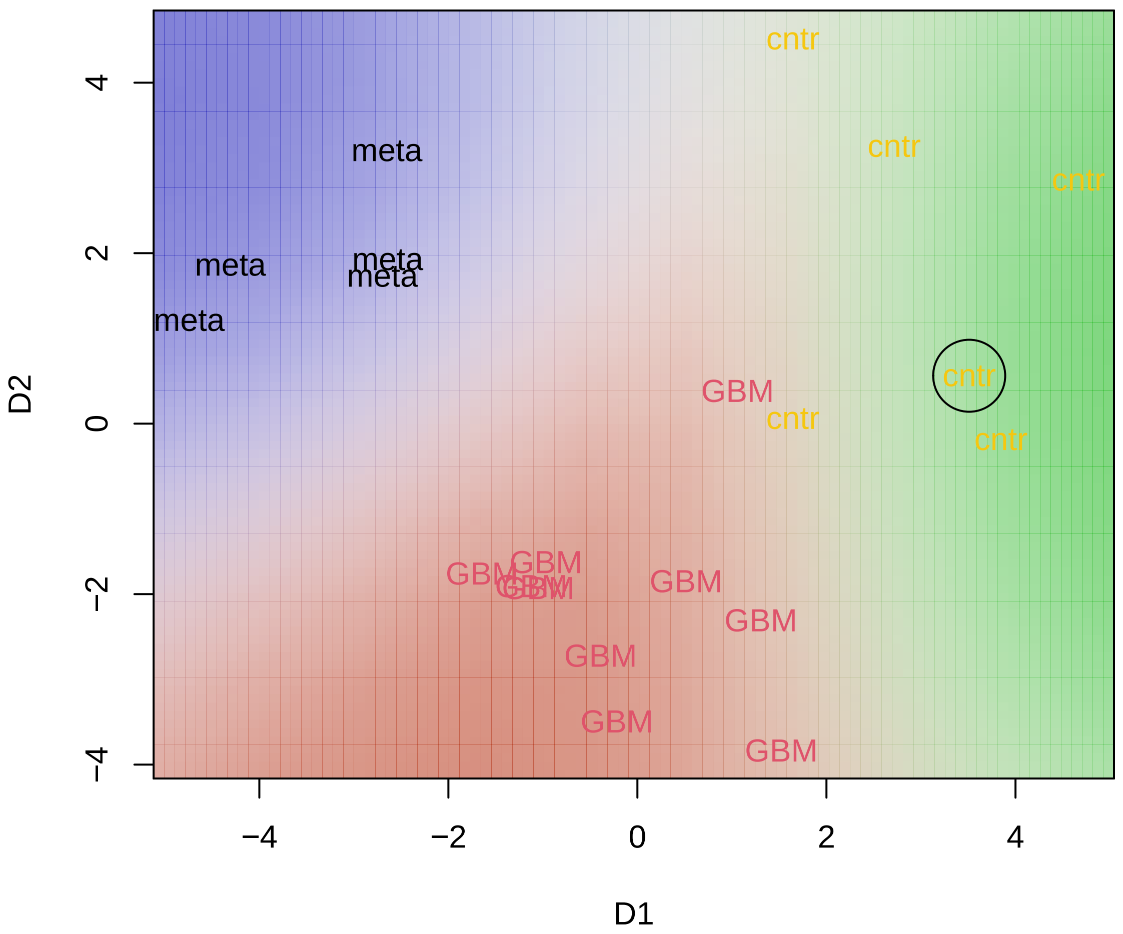

SVM_SupplFig_19.pdf

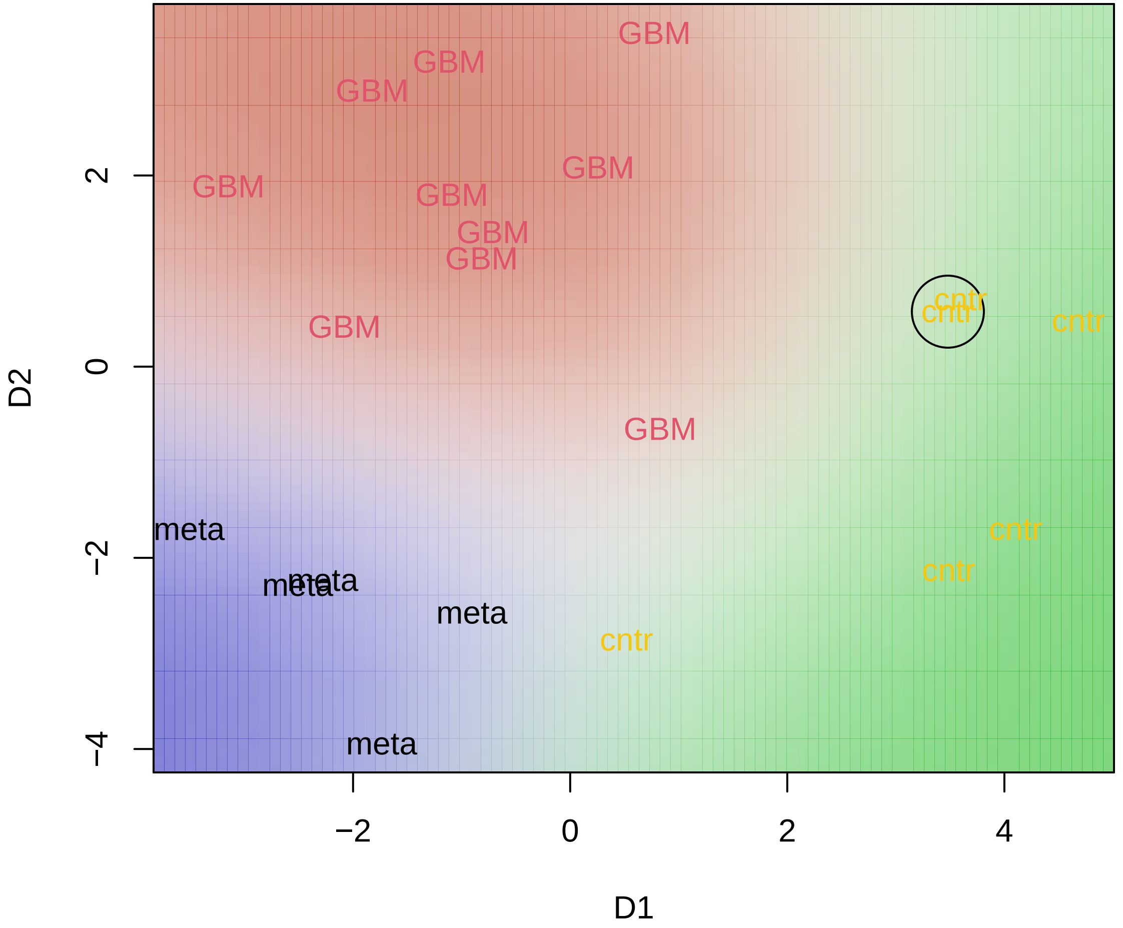

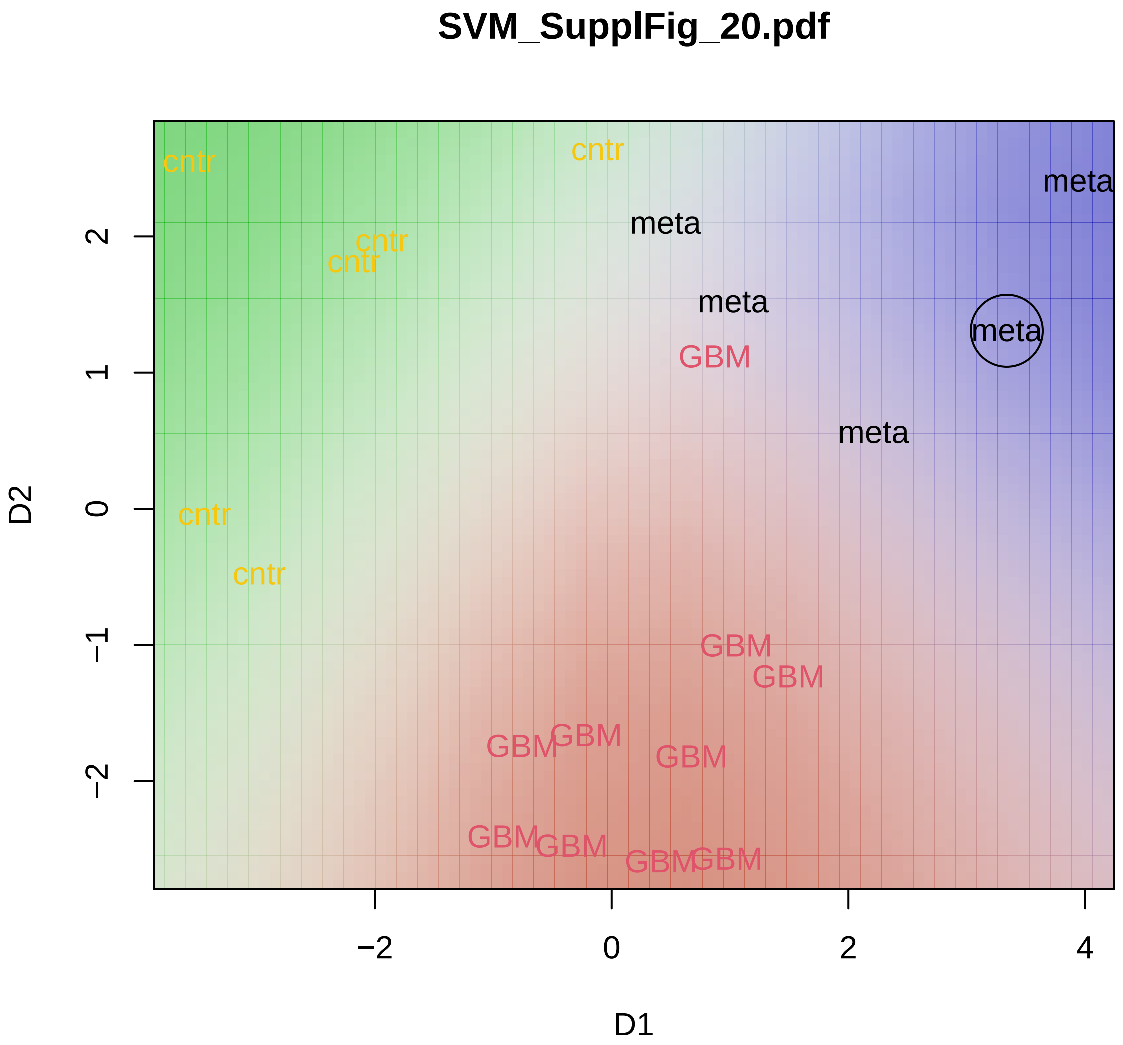
SVM_SupplFig_20.pdf
D1
D2
−2
0
2
4
−2
−1
0
1
2
cntr
cntr
cntr
cntr
cntr
cntr
meta
meta
meta
meta
meta
GBM
GBM
GBM
GBM
GBM
GBM
GBM
GBM
GBM
GBM

SVM_SupplFig_21.pdf

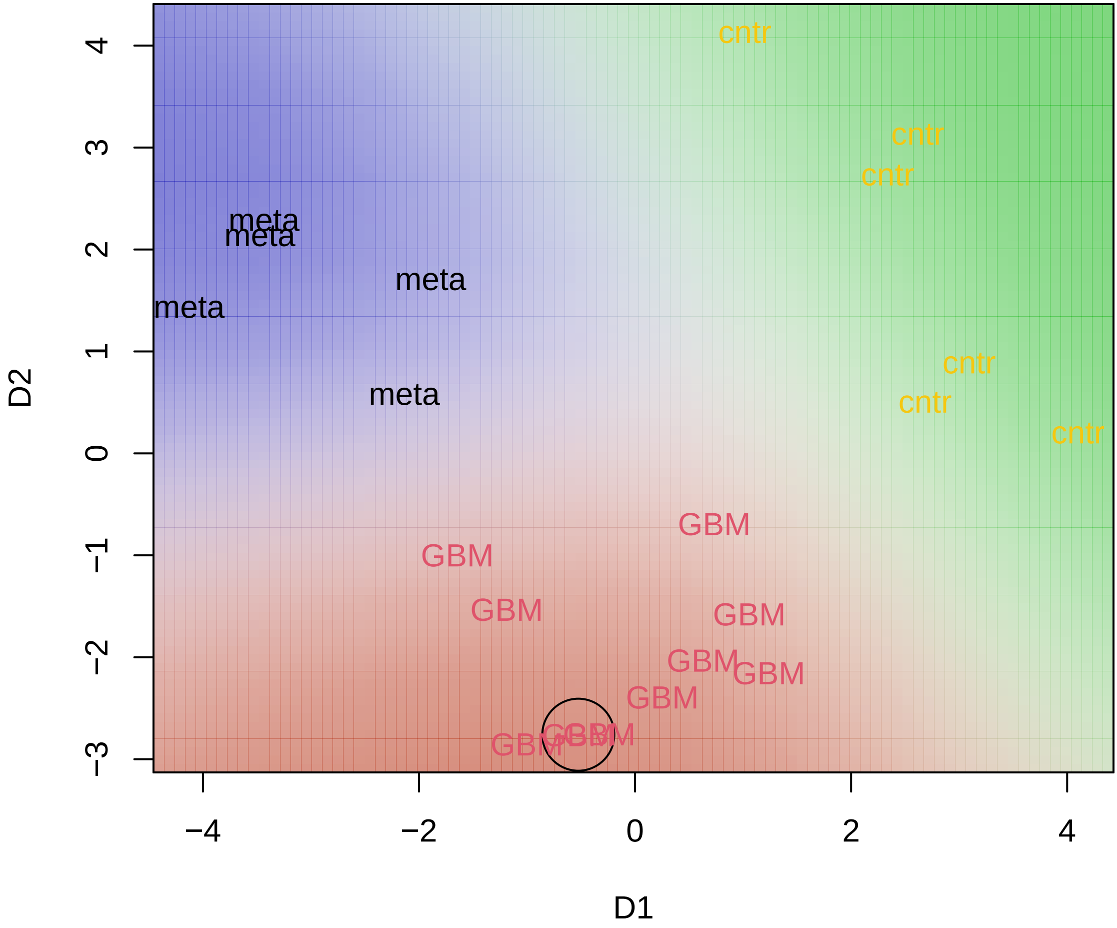

Supplement: Supplementary file 1 [file ijms-24-02597-s001.zip › SupplFigS8.pdf]
