# Supplementary material for: Reactivity Graph Yields Interpretable IgM Repertoire Signatures as Potential Tumor Biomarkers
Source: Int J Mol Sci. 2023 Jan 30;24(3):2597. doi: 10.3390/ijms24032597 (PMC9917253; doi:10.3390/ijms24032597)
Supplement: Supplementary file 1 [file ijms-24-02597-s001.zip › SupplFileS2/fimo_out_10/fimo.html]

FIMO Results


---

|  |  |  |  |  |
| --- | --- | --- | --- | --- |
| **Database and Motifs** | **High-scoring Motif Occurences** | **Debugging Information** | **Results in TSV Format** | **Results in GFF3 Format** |

  
  


---
