# Supplementary figures and images for: Reactivity Graph Yields Interpretable IgM Repertoire Signatures as Potential Tumor Biomarkers
Source: Int J Mol Sci. 2023 Jan 30;24(3):2597. doi: 10.3390/ijms24032597 (PMC9917253; doi:10.3390/ijms24032597)

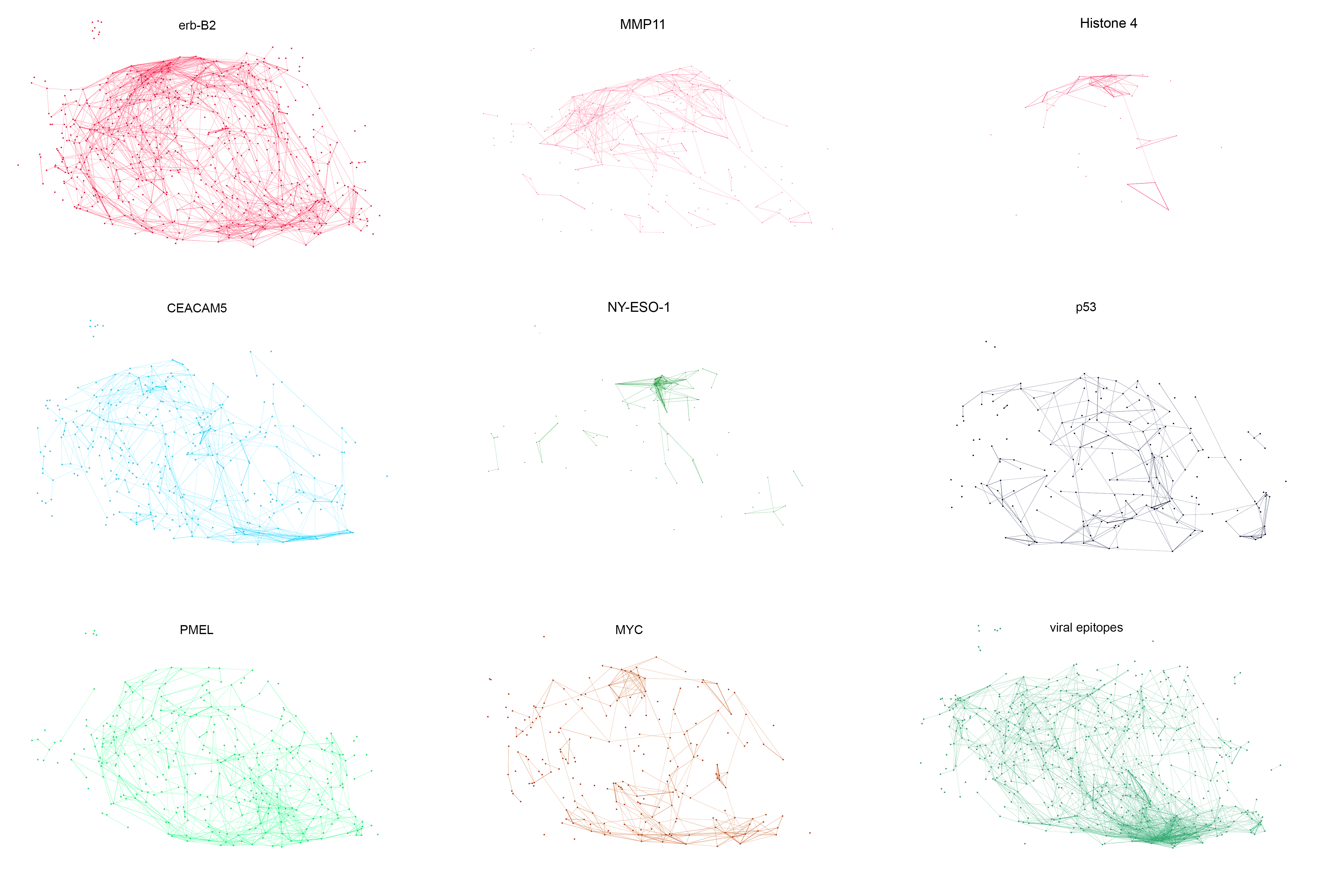

Supplement: Supplementary file 1 [file ijms-24-02597-s001.zip › SupplFigS2.png]

N

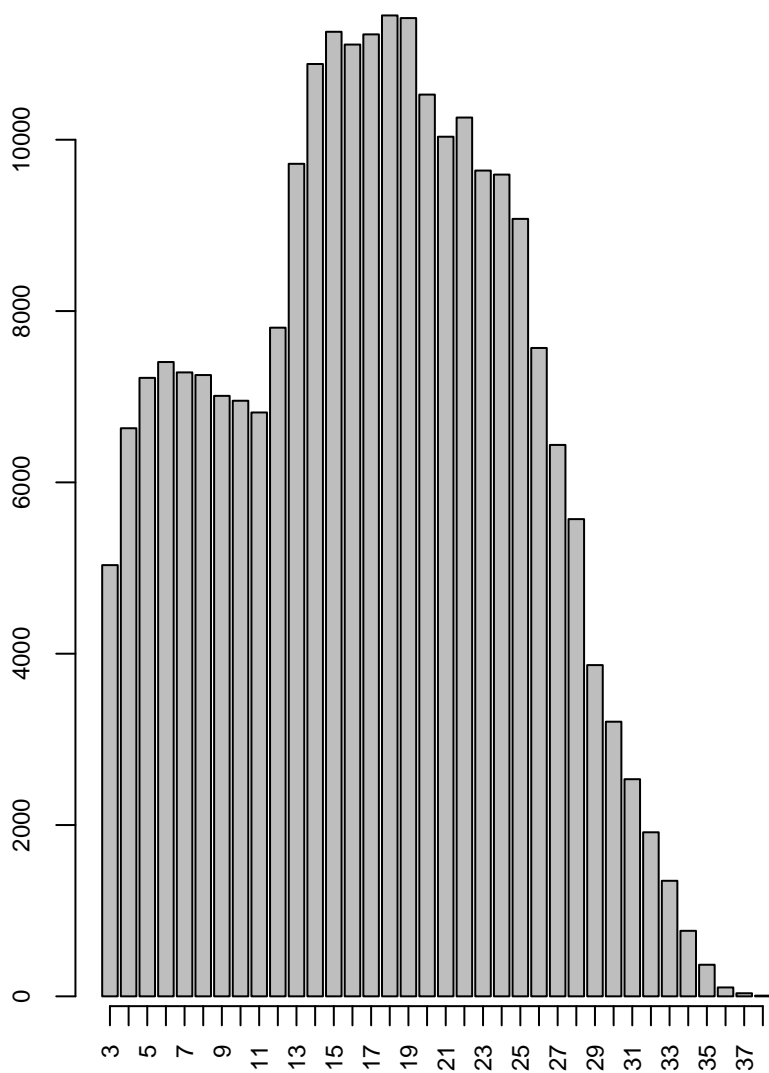

Clique Size

Supplement: Supplementary file 1 [file ijms-24-02597-s001.zip › SupplFigS3.pdf]

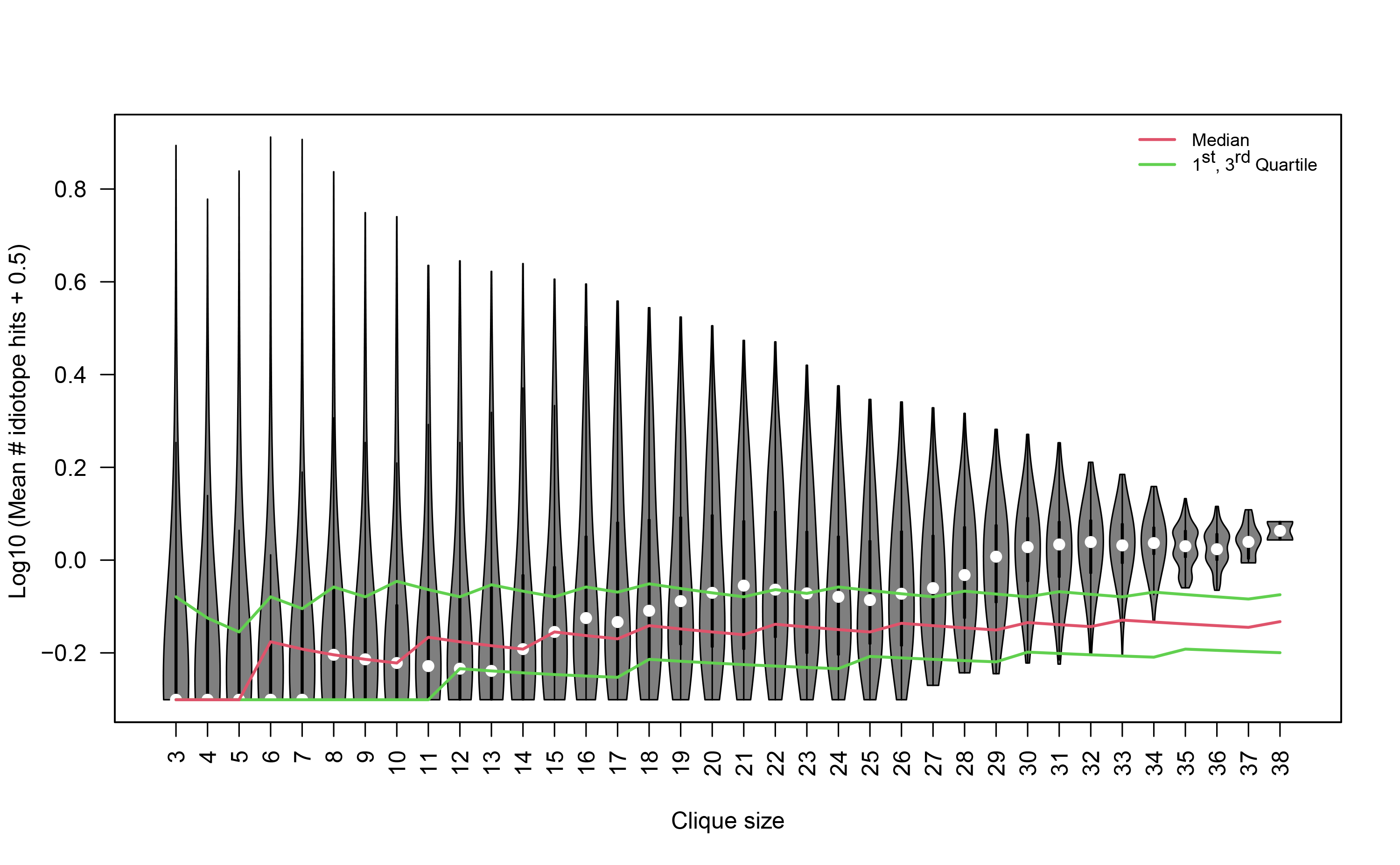

Supplement: Supplementary file 1 [file ijms-24-02597-s001.zip › SupplFigS4.tif]

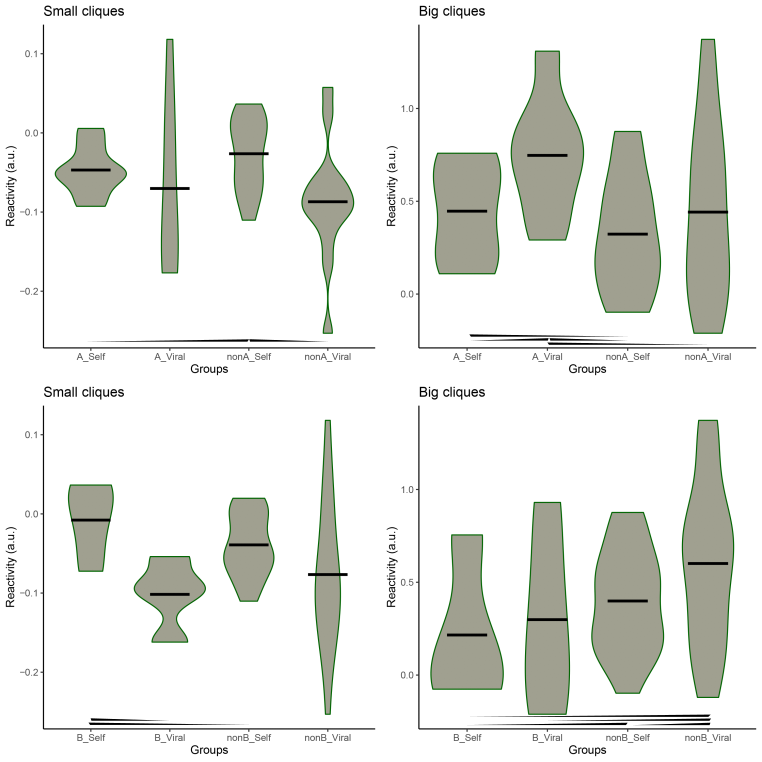

Supplement: Supplementary file 1 [file ijms-24-02597-s001.zip › SupplFigS5.tiff]

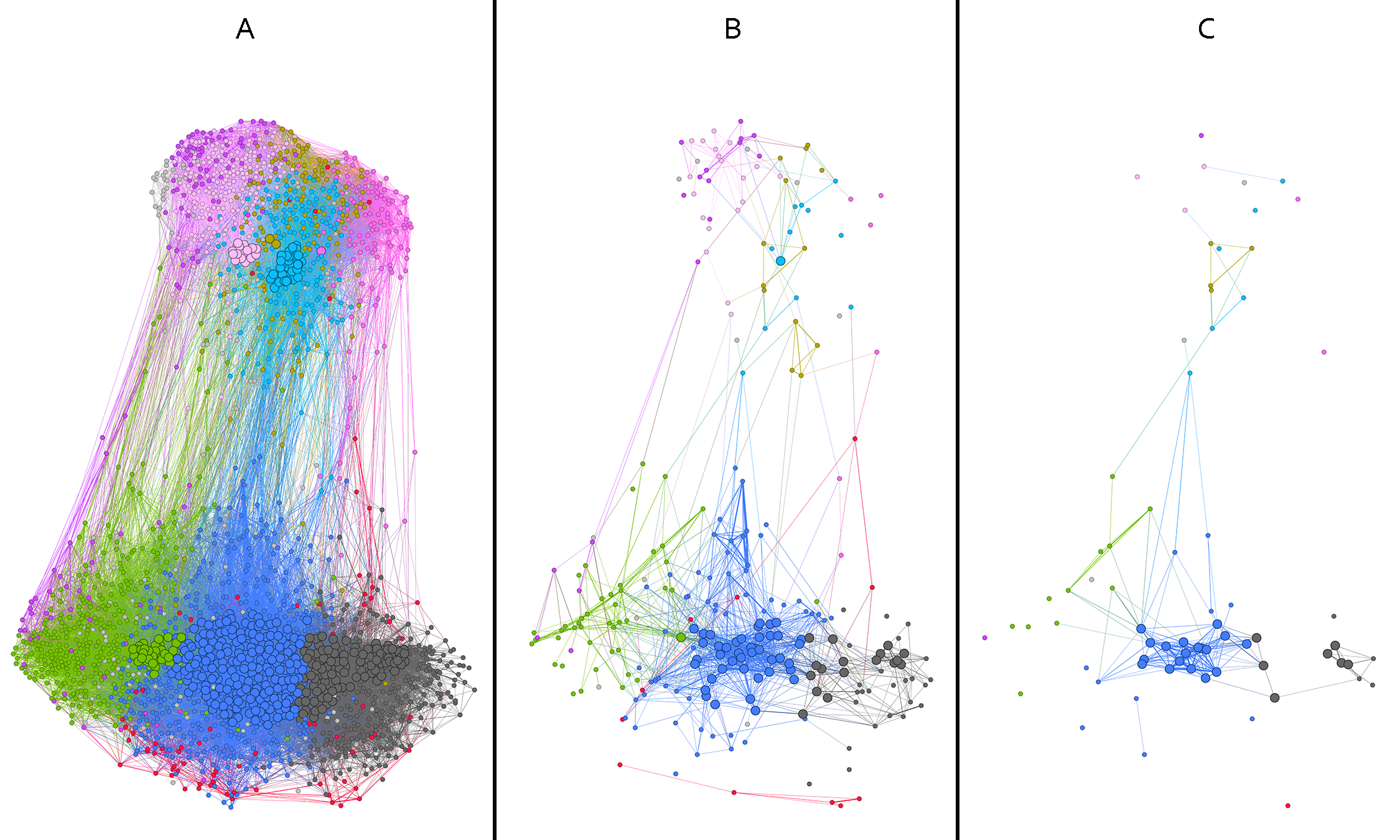

Supplement: Supplementary file 1 [file ijms-24-02597-s001.zip › SupplFigS6.tif]

Scree plot

A

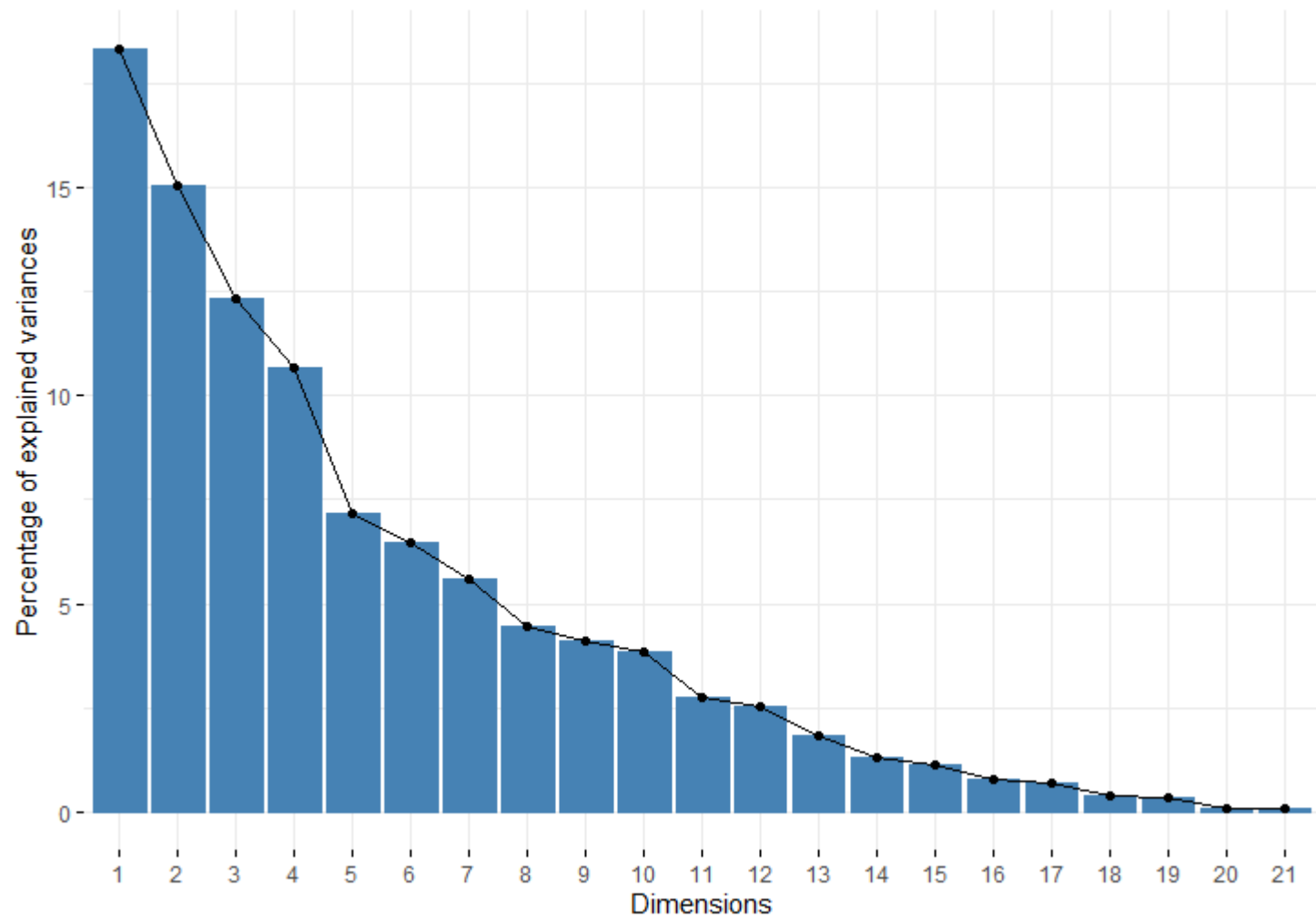

PCA - Biplot

B

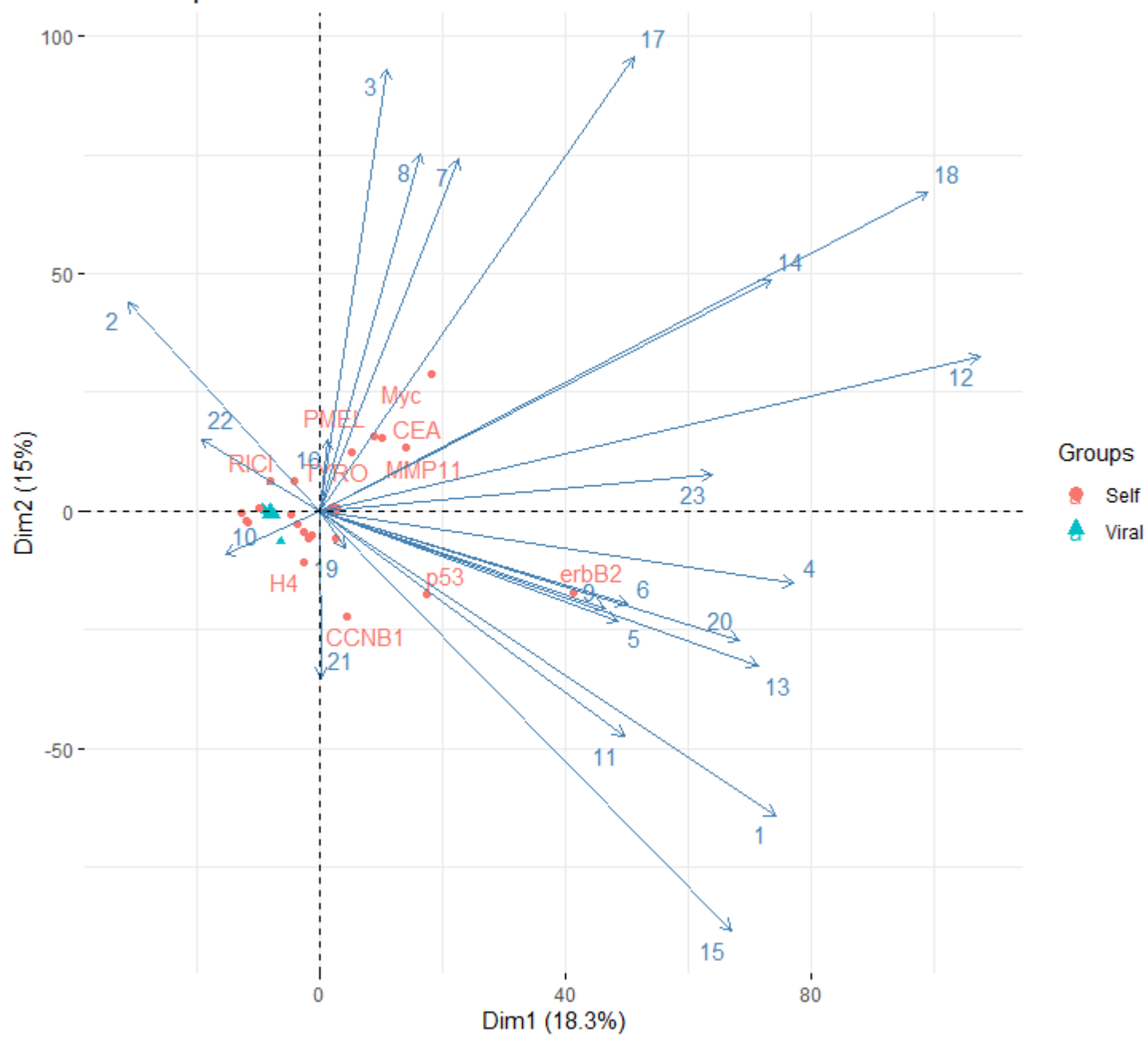

PCA - Biplot

C

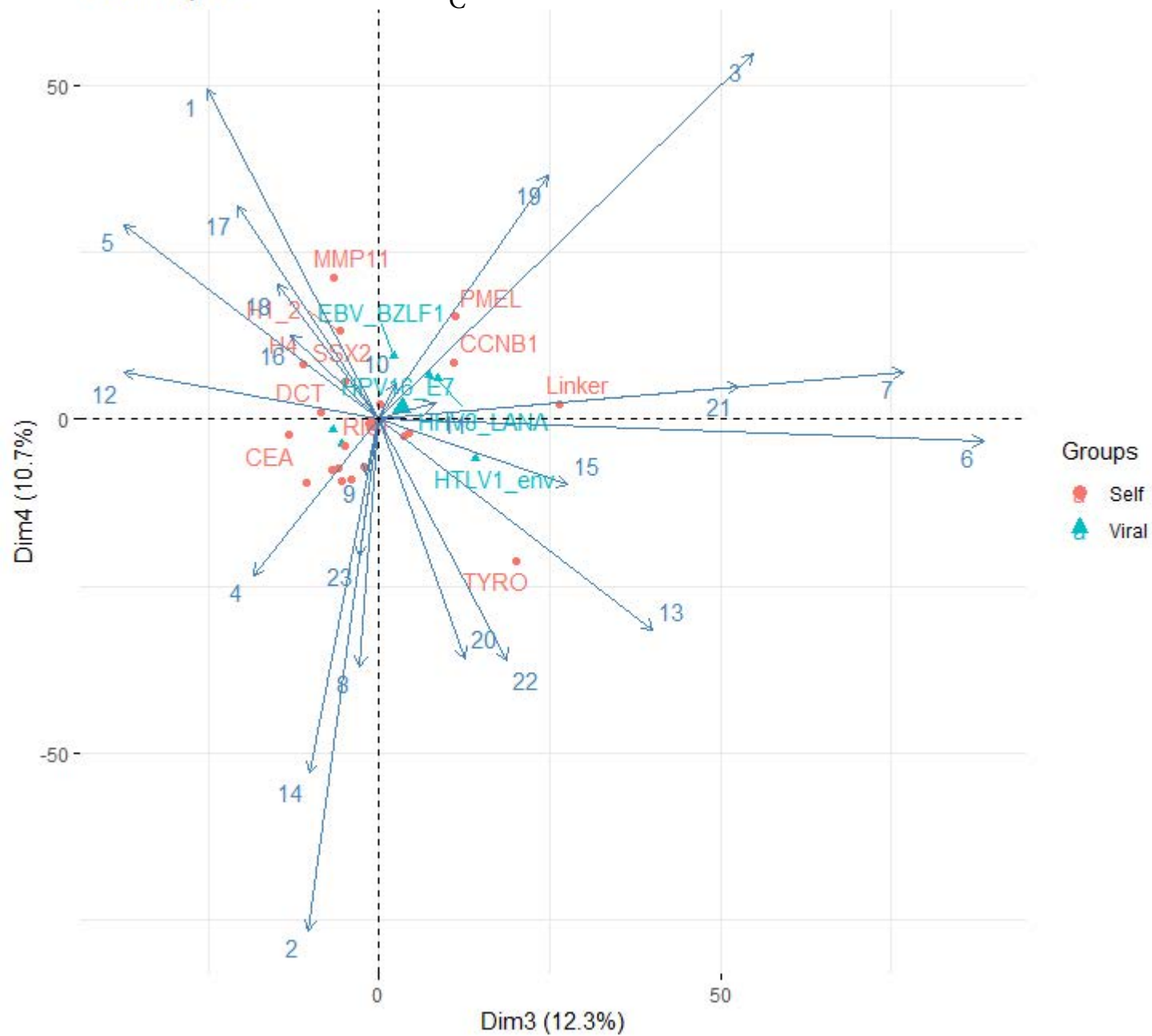

## PCA - Biplot

D

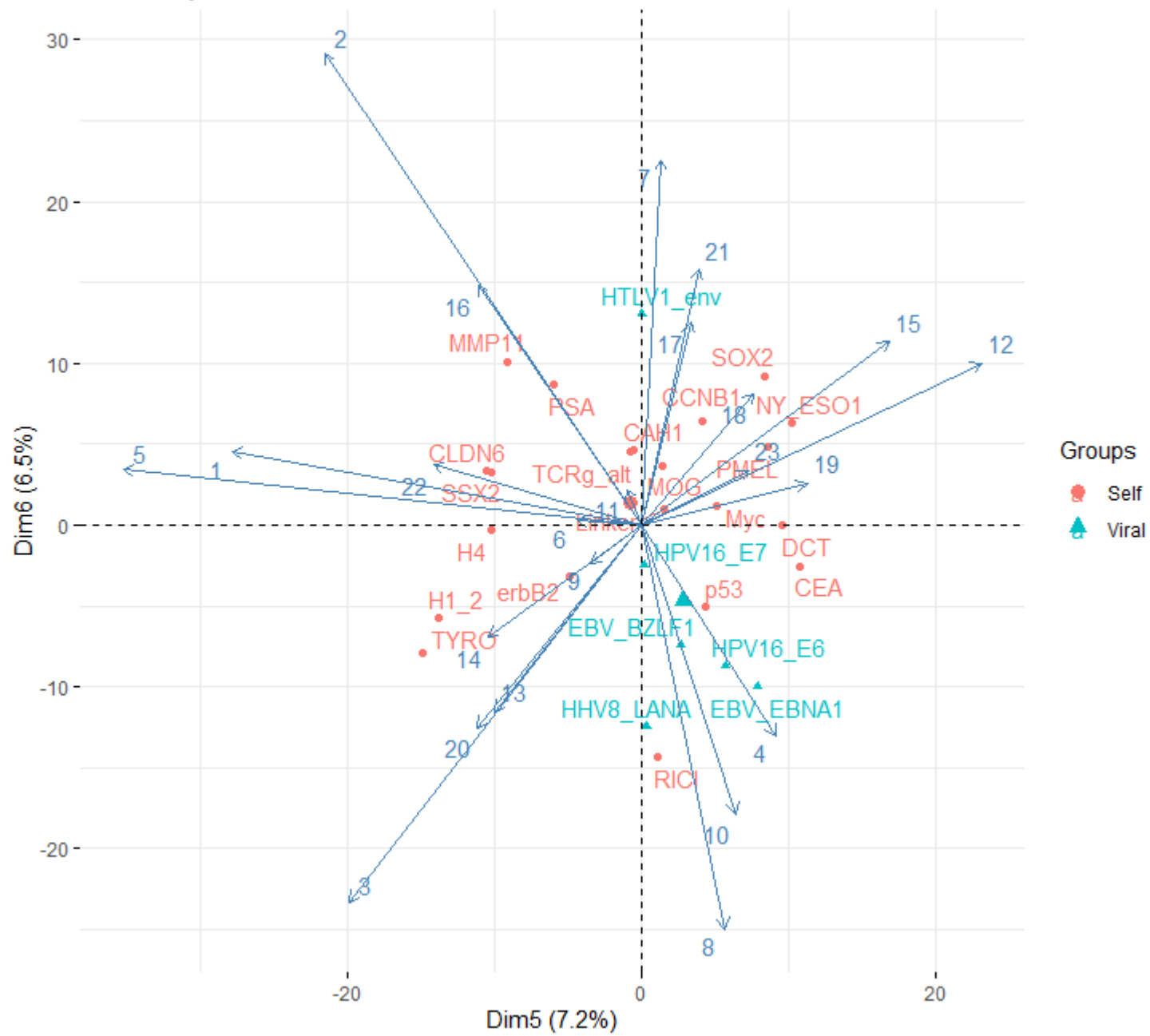

Supplement: Supplementary file 1 [file ijms-24-02597-s001.zip › SupplFigS7.pdf]

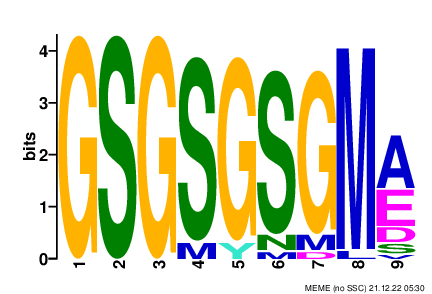

Supplement: Supplementary file 1 [file ijms-24-02597-s001.zip › SupplFileS2/meme_out/logo1.png]

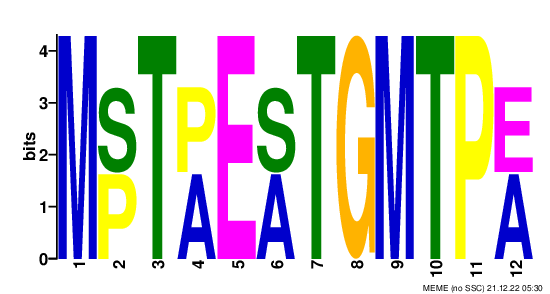

Supplement: Supplementary file 1 [file ijms-24-02597-s001.zip › SupplFileS2/meme_out/logo10.png]

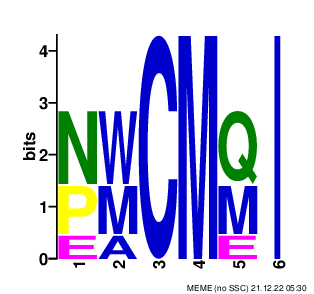

Supplement: Supplementary file 1 [file ijms-24-02597-s001.zip › SupplFileS2/meme_out/logo11.png]

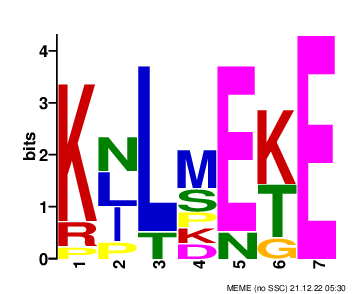

Supplement: Supplementary file 1 [file ijms-24-02597-s001.zip › SupplFileS2/meme_out/logo12.png]

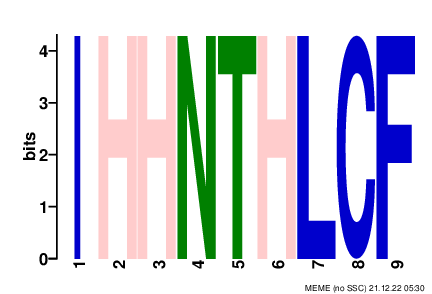

Supplement: Supplementary file 1 [file ijms-24-02597-s001.zip › SupplFileS2/meme_out/logo13.png]

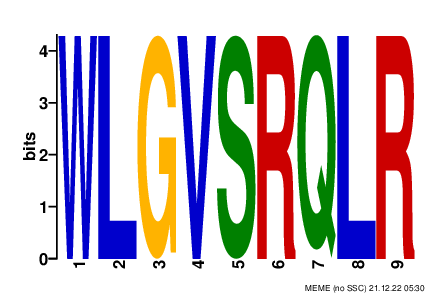

Supplement: Supplementary file 1 [file ijms-24-02597-s001.zip › SupplFileS2/meme_out/logo14.png]

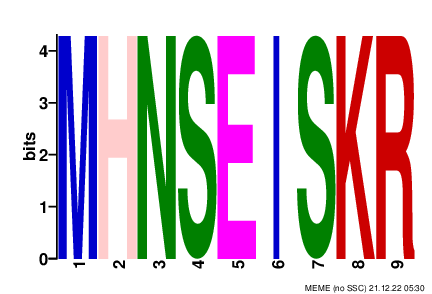

Supplement: Supplementary file 1 [file ijms-24-02597-s001.zip › SupplFileS2/meme_out/logo15.png]

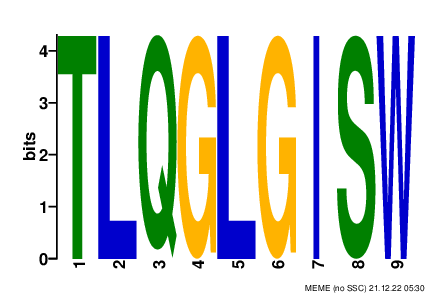

Supplement: Supplementary file 1 [file ijms-24-02597-s001.zip › SupplFileS2/meme_out/logo16.png]

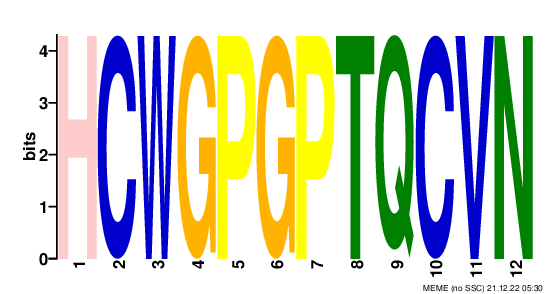

Supplement: Supplementary file 1 [file ijms-24-02597-s001.zip › SupplFileS2/meme_out/logo17.png]

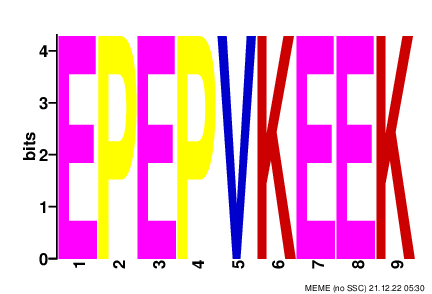

Supplement: Supplementary file 1 [file ijms-24-02597-s001.zip › SupplFileS2/meme_out/logo18.png]

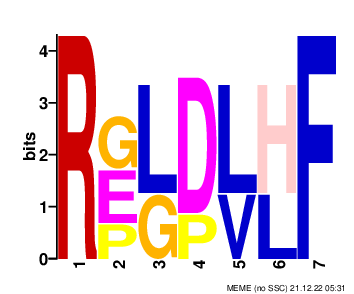

Supplement: Supplementary file 1 [file ijms-24-02597-s001.zip › SupplFileS2/meme_out/logo19.png]

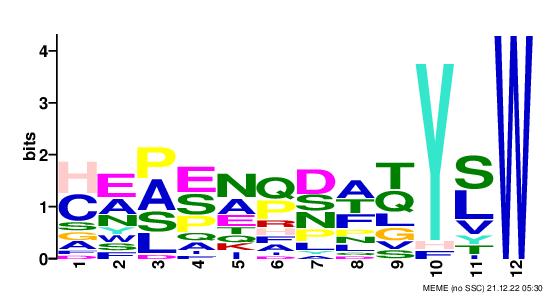

Supplement: Supplementary file 1 [file ijms-24-02597-s001.zip › SupplFileS2/meme_out/logo2.png]

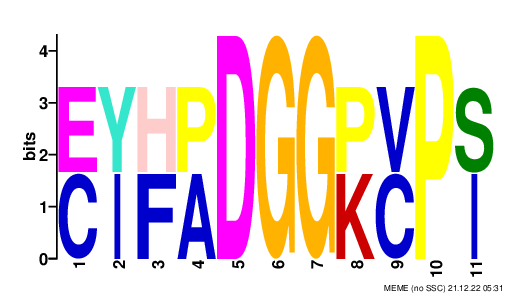

Supplement: Supplementary file 1 [file ijms-24-02597-s001.zip › SupplFileS2/meme_out/logo20.png]

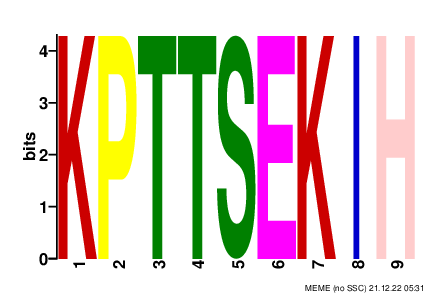

Supplement: Supplementary file 1 [file ijms-24-02597-s001.zip › SupplFileS2/meme_out/logo21.png]

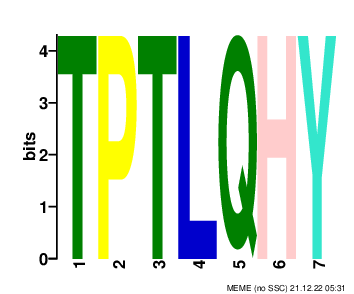

Supplement: Supplementary file 1 [file ijms-24-02597-s001.zip › SupplFileS2/meme_out/logo22.png]

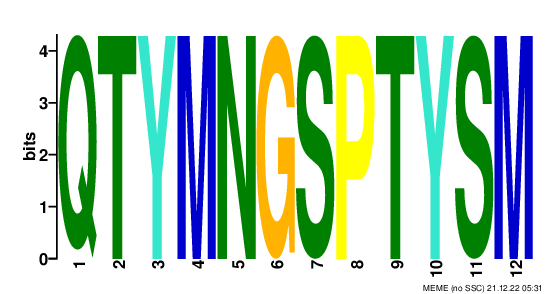

Supplement: Supplementary file 1 [file ijms-24-02597-s001.zip › SupplFileS2/meme_out/logo23.png]

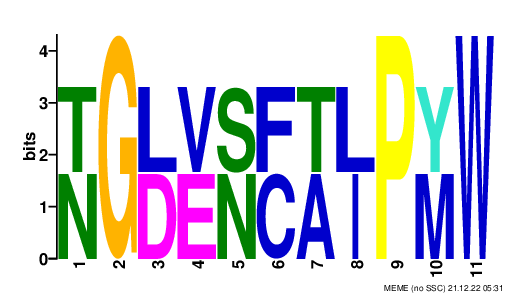

Supplement: Supplementary file 1 [file ijms-24-02597-s001.zip › SupplFileS2/meme_out/logo24.png]

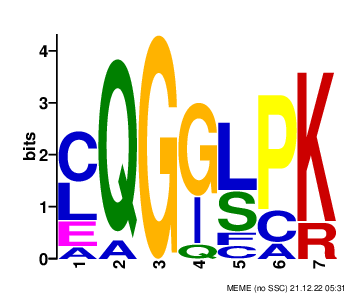

Supplement: Supplementary file 1 [file ijms-24-02597-s001.zip › SupplFileS2/meme_out/logo25.png]

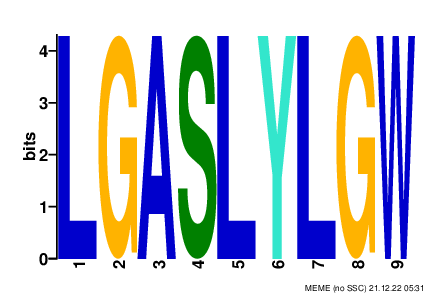

Supplement: Supplementary file 1 [file ijms-24-02597-s001.zip › SupplFileS2/meme_out/logo26.png]

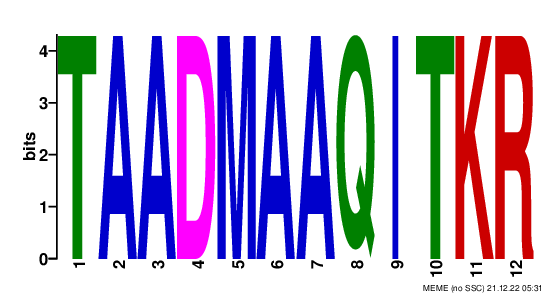

Supplement: Supplementary file 1 [file ijms-24-02597-s001.zip › SupplFileS2/meme_out/logo27.png]

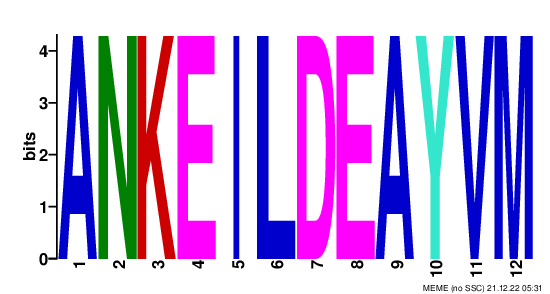

Supplement: Supplementary file 1 [file ijms-24-02597-s001.zip › SupplFileS2/meme_out/logo28.png]

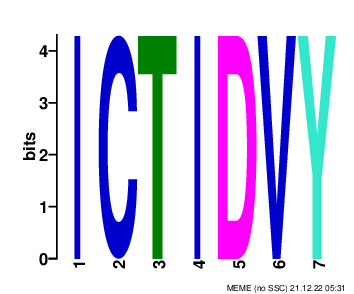

Supplement: Supplementary file 1 [file ijms-24-02597-s001.zip › SupplFileS2/meme_out/logo29.png]

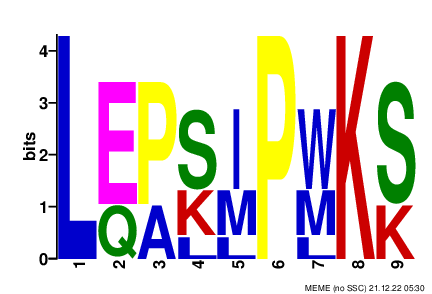

Supplement: Supplementary file 1 [file ijms-24-02597-s001.zip › SupplFileS2/meme_out/logo3.png]

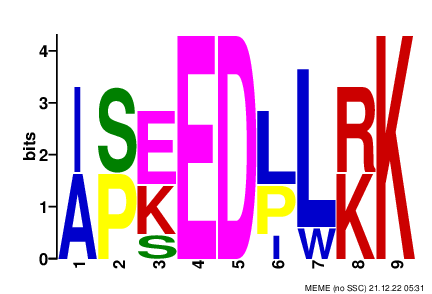

Supplement: Supplementary file 1 [file ijms-24-02597-s001.zip › SupplFileS2/meme_out/logo30.png]

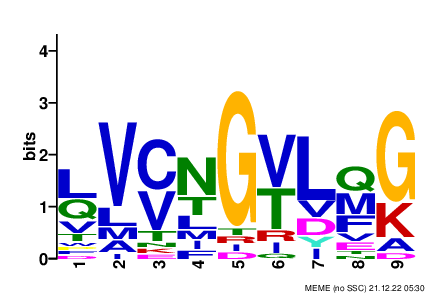

Supplement: Supplementary file 1 [file ijms-24-02597-s001.zip › SupplFileS2/meme_out/logo4.png]

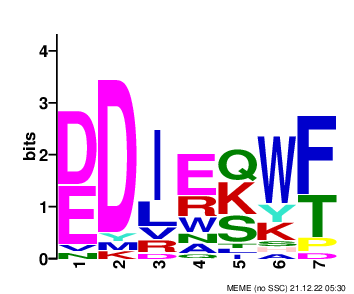

Supplement: Supplementary file 1 [file ijms-24-02597-s001.zip › SupplFileS2/meme_out/logo5.png]

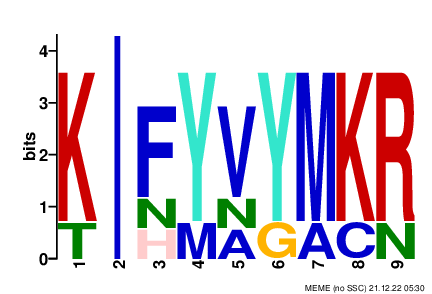

Supplement: Supplementary file 1 [file ijms-24-02597-s001.zip › SupplFileS2/meme_out/logo6.png]

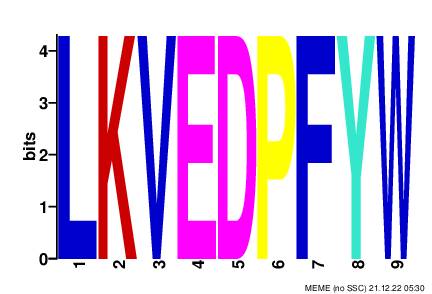

Supplement: Supplementary file 1 [file ijms-24-02597-s001.zip › SupplFileS2/meme_out/logo7.png]

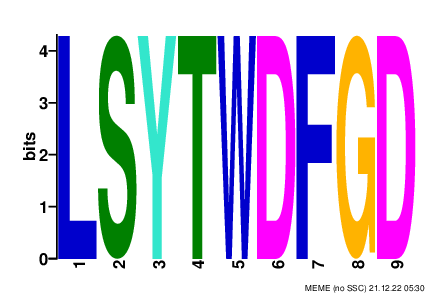

Supplement: Supplementary file 1 [file ijms-24-02597-s001.zip › SupplFileS2/meme_out/logo8.png]

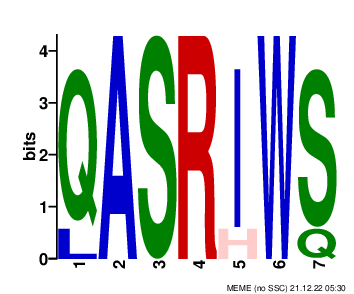

Supplement: Supplementary file 1 [file ijms-24-02597-s001.zip › SupplFileS2/meme_out/logo9.png]
