# Supplementary material for: Reactivity Graph Yields Interpretable IgM Repertoire Signatures as Potential Tumor Biomarkers
Source: Int J Mol Sci. 2023 Jan 30;24(3):2597. doi: 10.3390/ijms24032597 (PMC9917253; doi:10.3390/ijms24032597)
Supplement: Supplementary file 1 [file ijms-24-02597-s001.zip › SupplLegends.pdf]

Suppl. Figure S1. Correlations between the patients' IgM reactivities with the array of peptides.

Suppl. Figure S2. Mapping of 8 of the 24 studied human protein TAA as well as all viral epitopes on the reactivity graph. No particular segregation for large enough proteins, indicating that the reactivity map is based on local chemical/structural features of the linear epitopes rather than on the (induced) immune reactivity to the whole antigen. There is preference for some viral epitopes in cluster 6 (see Suppl. Fig. 9).

Suppl. Figure S3. Distribution of the number of maximal cliques in the reactivity graph by size.

Suppl. Figure S4. Violin plot - distribution of the mean number of potential idiotopes (J region sequences) found in the tested antigens as a function of the clique size. Lines – mean, 1st and 3rd quartile of the distributions calculated on the basis of 5000 random peptide samples of the size of the respective cliques. Big cliques have disproportionately more potential idiotope sequences.

Suppl. Figure S5. Violin plots of the distributions of the reactivity in small and big cliques as a function of blood group antigen expression of the patient and viral or self nature of the epitope. Viral epitopes have higher reactivity in big cliques and lower reactivity in small cliques than self epitopes and this is more pronounced in blood group A patients. The significant differences in the means are marked by lines bellow the plots.

Suppl. Figure S6. Induced subgraph of the reactivity graph based on the ego 1 network (small circles) of the vertices in the big cliques (large circles). A – All vertices, the layout is chosen to correspond approximately to the position of the vertices in the starting reactivity graph and the colors correspond to the coloring of the clusters in the reactivity graph (Fig.7). B – Induced subgraph of only the viral epitopes in A and their ego network. C - Induced subgraph of the HTLV1 epitopes in B and their ego network.

Suppl. Figure S7. A set of plots from principal component analysis of the reactivities to the peptides included in the potentially diagnostic cliques and graph GpClq. The contribution of the different antigens to the reactivities in GpClq by clique is visualized. A – A scree plot illustrating the contribution to the overall variance of the extracted principal components. B, C and D – the biplots of, respectively, the 1st and 2nd, 3rd and 4th and 5th and 6th component.

Suppl. Figure S8. Visualizations of the "leave one out" cross-validation based on support vector machine models on 2-dimensional embeddings of the data based on features selected using the reactivity graph and recursive feature elimination. Each plot represents the results for the classification of a single case based on the classifier constructed on the remaining 20 cases. The classified (left out) case is encircled.
